# Genome-Scale Analysis of Perturbations in Translation Elongation Based on a Computational Model

**DOI:** 10.1038/s41598-018-34496-3

**Published:** 2018-11-01

**Authors:** Doron Levin, Tamir Tuller

**Affiliations:** 1 Department of Biomedical Engineering, the Engineering Faculty, Tel Aviv University, Tel-Aviv, 69978 Israel; 2 The Sagol School of Neuroscience, Tel-Aviv University, Tel-Aviv, 69978 Israel

## Abstract

Perturbations play an important role both in engineered systems and cellular processes. Thus, understanding their effect on protein synthesis should contribute to all biomedical disciplines. Here we describe the first genome-scale analysis of perturbations in translation-related factors in *S. cerevisiae*. To this end, we used simulations based on a computational model that takes into consideration the fundamental stochastic and bio-physical nature of translation. We found that the initiation rate has a key role in determining the sensitivity to perturbations. For low initiation rates, the first codons of the coding region dominate the sensitivity, which is highly correlated with the ratio between initiation rate and mean elongation rate (*r* = −0.95), with the open reading frame (ORF) length (*r* = 0.6) and with protein abundance (*r* = 0.45). For high initiation rates (that may rise, for example, due to cellular growth), the sensitivity of a gene is dominated by all internal codons and is correlated with the decoding rate. We found that various central intracellular functions are associated with the sensitivity: for example, both genes that are sensitive and genes that are robust to perturbations are over-represented in the group of genes related to translation regulation; this may suggest that robustness to perturbations is a trait that undergoes evolutionary selection in relation to the function of the encoded protein. We believe that the reported results, due to their quantitative value and genome-wide perspective, should contribute to disciplines such as synthetic biology, functional genomics, comparative genomics and molecular evolution.

## Introduction

mRNA translation is a central gene expression step that occurs in all living organisms^1^. Thus, understanding, modelling, and engineering this process have important ramifications to every biomedical discipline, including molecular evolution and comparative genomics^2,3^, medicine and human health^4,5^, biotechnology^6^ agriculture^7^ and more. The amount of intracellular resources related to translation (such as ribosomes, tRNAs, elongation factors, enzymes etc.) is constantly changing in response to various regulatory mechanisms and phenomena, e.g. processes which are driven by circadian clock. These changes (or perturbations) are rarely modeled, and their effect on the translation rate (and consequently, on gene expression) is poorly understood.

The study of the effect of perturbations on endogenous and heterologous expression levels is a fundamental research topic that should enable the understanding of the evolution of genes and genomes, their comparison^8–16^ and other related phenomena. Furthermore, since clocks (that cause inevitable perturbations due to oscillatory behavior) are central components in most engineered systems, they are expected to appear also in synthetic intracellular systems^17–20^. In addition, various recent studies have emphasized the contribution of translation to intracellular oscillations and fluctuations^10,14,16,21^, which are naturally related to perturbations.

**Figure 1:**
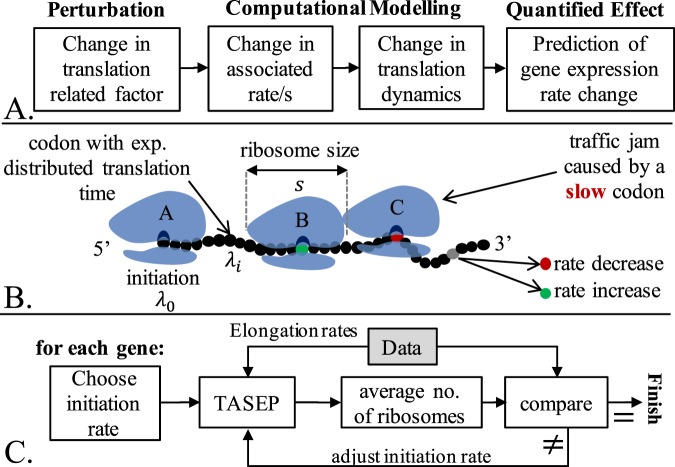
(**A**) A schematic description of the underlying approach: a perturbed factor is chosen to account for some type of biological perturbation (e.g. decrease in a given type of tRNA); the effect of the perturbation is modeled using a dynamic model, leading to a change in the translation dynamics; finally, a quantified gene expression change is obtained. (**B**) An illustration of TASEP in the elongation context; the ribosome *C* is delayed due to translation of a “slow codon” (e.g. with low tRNA abundance), forcing the trailing ribosome *B* to stall. Ribosome *A* is near the 5′ end of the ORF and has recently performed initiation. The rate of each codon can be modified to model the desired perturbation. (**C**) Flow diagram of the initiation rate estimation procedure. This procedure is performed separately for each gene to ensure proper local initiation time. We first arbitrarily choose a positive initiation rate and then iteratively improve agreement between estimated and reported (by Arava *et al*.^34^) average number of ribosomes. At each step, a TASEP simulation is performed (using gene-specific elongation rates and the chosen initiation rate), and the resulting number of ribosomes is compared to experimental data. The initiation rate is tuned until the result fits the experimental data with up to 2% error.

Codon decoding rates and initiation rates are key factors in understanding and modelling the effect of cellular perturbations on mRNA translation. On one hand, these rates are deeply associated with various intracellular “resources” that are affected by the state of the cell (e.g. ribosome or tRNA pool, elongation factors, enzymes etc.). On the other hand, these rates can greatly alter elongation dynamics. As an example, consider a change of specific aminoacyl tRNA type (a behavior that was recently linked to cancer^22^), which in turn affects all decoding rates of codons associated with those tRNA molecules. These changes may disrupt mRNA translation, for example, by creating ribosomal ‘traffic jams’. Our general methodology, which is schematically described in Fig. 1A, is based on a computational model that predicts the effect of such perturbations. A suitable model (to be described below), allows changing these rates in a customized way and predicting the change in the translation rates. It should be mentioned that the studied perturbations are assumed not to affect the assembly of the pre-initiation complex, as explained in the Discussion section.

A previous study was based on a mathematical analysis of simple deterministic models and was not based on genomic data (e.g.^19^). Furthermore, currently there are no practical experimental tools that can be easily used to perform an accurate, high resolution analysis of perturbations in translation-related factors for the entire *S. cerevisiae* genome: Specifically, it is extremely difficult to design and perform a reliable experiment that properly isolates the discussed effect for each codon in each gene at a single-cell level. To bridge these gaps, our study aims at creating a genome-scale framework for quantifying the effect of perturbations. Specifically, our approach includes performing accurate simulations using a computational model that takes into consideration the bio-physical nature of the translation process (e.g. stochasticity and interactions between ribosomes), while perturbing various translation-related factors (that affect the associated rates). We used parameters inferred based on biological measurements including ribosome size, typical codon decoding rates, and analyzed real genes from the *S. cerevisiae* genome. Thus, our study is an important step towards understanding and accurate modelling of translation perturbations at a genomic level.

## Methods

### The Constant Perturbations Approximation

Typically, elongation rate is five to ten codons per second, i.e. a coding region that consists of few hundred codons is expected to be translated within seconds to very few minutes^23^. On the other hand, if periodic changes in translation-related factors are considered, we assume here that these changes (e.g. changes in the expression levels of tRNAs) are expected to take at least an order-of-magnitude longer. This justifies an adoption of a simple approach for studying the effect of constant perturbations in different factors, which in turn can serve as a decent approximation for slow time-dependent fluctuations analysis. This work is focused on biological changes that can be modeled as time-independent changes in codon decoding rates, which are the parameters of the model described below.

### Computational Model of mRNA Translation

The elongation step of mRNA translation involves stochastic propagation of ribosomes and is traditionally modelled and analyzed using the Totally Asymmetric Exclusion Process (TASEP)^24–26^. This approach incorporates local elongation rates and unidirectional movement (5′ to 3′) of ribosomes with physical size (of *s* codons), that are not allowed to overlap or overtake one another, resulting in possible ‘traffic jams’ (see Fig. 1B and Supplementary Methods S1 for additional details). The ribosome size is denoted by *s* and was chosen to be 9 codons throughout all simulations^27^, although similar results were obtained when using 11 or 13 codons (see Supplementary Methods S2 and Fig. S1).

### Translation Initiation and Elongation Rates

#### Local Initiation Rate

Initiation rate is a function of physical features such as the number of available free ribosomes^28,29^, the folding energy at the 5′ end of the coding sequence, the base pairing potential between the 5′ UTR and the ribosomal rRNA (in prokaryotes), and other properties of the 5′ UTRs^28,30–33^. In our study, the initiation rate was estimated for each mRNA separately based on ribosome densities^34^ (obtained from polysome profiling by microarray analysis for almost all *S. cerevisiae* genes). As schematically described in Fig. 1C, we tuned the initiation rate until the predicted average number of ribosomes on the mRNA was equal to the values reported by Arava *et al*.^34^ (see Supplementary Methods S3 and Fig. S2). It is important to note that initiation rate here is defined as an effective rate, incorporating all relevant mechanisms. The initiation rate of a mRNA is denoted by *λ*_0_.

We will refer to the estimated initiation rates as the default or ‘baseline’ values. It is known that there can be extreme changes in the number of ribosomes and other translation-related factors at different conditions and growth rates^9,35^, resulting in corresponding changes in the ratio between elongation and initiation rates. In addition, in the case of synthetic systems, the initiation rates of mRNAs can be designed to be extremely high. To account for these scenarios, we also analyzed the system when all the baseline initiation rates were multiplied by a factor of up-to 10. This increased factor is expected to reveal all the expected behavior in terms of initiation rate. Moreover, it was important to work with a single global factor in order to preserve the relative values within the distribution of initiation rate values. The factor at which the baseline initiation rate is multiplied by is denoted by *α*.

#### Codon Decoding Rates

The translation process is generally considered more initiation rate limited than elongation rate limited^36^. However, it has been shown that, in many cases, codon-specific elongation rates can be crucial for accurate prediction of translation dynamics and rate^33,37,38^. To demonstrate this, we calculated a ‘bottleneck factor’, defined as the ratio between initiation rate and the minimum effective elongation rate of any *s* codons in the ORF. The resulting average value is 0.22, with more than 20% of the ORFs having values larger than 0.25. This emphasizes the importance of incorporating codon decoding rates into the model. See Supplementary Methods S4 for more details.

The typical decoding rate (TDR) for each codon was computed in^39^. These estimations are based on ribosome profiling (or Ribo-Seq)^40^, while considering and filtering biases, pauses, ‘traffic jams’, etc. For a given coding region, we define $\underline{\lambda }$ = (*λ*_1_, *λ*_2_, …, *λ*_*n*_), where *n* is the length of the gene ORF in codons and *λ*_*i*_ is the TDR of the *i*^*th*^ codon. With this definition, the decoding time of codon *i* is assumed to be distributed exponentially^41,42^, namely $\sim \,\exp ({\lambda }_{i}^{-1})$.

### Modelling Perturbations and Translation Rate Sensitivity

We model a perturbation as a change in the initial parameters of the decoding rates vector $\underline{\lambda }$ and initiation rate *λ*_0_. The perturbation function $(\lambda {^{\prime} }_{0},\underline{\lambda }^{\prime} )=\theta ({\lambda }_{0},\underline{\lambda };\alpha ,\,p)$ returns a new initiation rate $\lambda {^{\prime} }_{0}=\alpha {\lambda }_{0}$ and a decoding rates vector, with an induced perturbation *p* at some location/s. We focus on two general types of perturbations, modelling a wide range of biological phenomena: (1) Location based perturbations, which alter the rate of specific codons (or several codons) at a specified location *i* in the mRNA. Such perturbations model local mechanical changes or local/short changes in resources abundance; (2) Global perturbations, related to all codons of a given type (e.g. codons that are recognized by a tRNA whose abundance has been altered).

Translation rate (which is equivalent to termination rate) is of great interest, so we would like to study how it is affected by perturbations. For a given $\underline{\lambda }$ and *λ*_0_, we define the translation rate *TR*_*τ*_(*λ*_0_, $\underline{\lambda }$) as the number of terminations divided by time segment *τ*. We calculate translation rate only after the stochastic process reaches steady-state (a procedure further discussed in Supplementary Methods S1), and we denote its mean value by *TR*(*λ*_0_, $\underline{\lambda }$). The translation rate sensitivity is defined per perturbation function *θ*:

1 $$sensitivity\triangleq \frac{\langle TR(\theta ({\lambda }_{0},\underline{\lambda };\alpha ,\,p))\rangle -\langle TR({\lambda }_{0},\underline{\lambda })\rangle }{\langle TR({\lambda }_{0},\underline{\lambda })\rangle }.$$

For example, to model a perturbation at codon *i*, we may define $\underline{\lambda }^{\prime}$ so that $\lambda {^{\prime} }_{i}(p)=(1+p)\,{\lambda }_{i}$. As another example, consider a perturbation at every codon *CTG* with initiation rate that is 5 times larger than the baseline. In this case, $\lambda {^{\prime} }_{0}=5{\lambda }_{0}$ and $\lambda {^{\prime} }_{i}(p)=(1+p)\,{\lambda }_{i}$ if the *i*^*th*^ codon of the ORF is *CTG*. If a perturbation was induced and sensitivity was calculated at each location *i* of the ORF, sensitivity will be referred to as *sensitivity profile* (i.e. index dependent), generally denoted as *SP*(*i*, *p*, *α*). ${\langle SP(i,\,p,\alpha )\rangle }_{i=a}^{b}$ is the average value of *SP* in the range *i* = *a*, …, *b*. The discussed sensitivity profile itself is usually an average profile of a group of genes. Specifically, the quantity ${\langle SP(i,p,\alpha )\rangle }_{i=1}^{9}$ (the average sensitivity of first 9 codons) will be discussed several times in the following text, so we will use the more intuitive notation ‘start region sensitivity’. When all codons are considered, namely ${\langle SP(i,p,\alpha )\rangle }_{i=1}^{n}$ (here and from now on, *n* denotes the last codon of each discussed coding region), we use the notation ‘overall sensitivity’. For convenience, we use percentage-based notation of *p*, so that, for example, *p* = 100% (−50%) means increasing (decreasing) the rate two-fold.

### Perturbation Magnitude

As mentioned above, the model analyzed here (TASEP) is a stochastic model of translation. The translation rate variability originated from the stochastic nature of TASEP may be larger than the simulated effect (i.e. the perturbations), masking the actual perturbations. In order to measure the sensitivity to perturbations reliably, we used an increase or decrease of 50% in the discussed rates. Nevertheless, we also tested how the main results may change due to different perturbation magnitudes. In particular, we showed how one can easily interpolate the expected effect of lower or higher perturbation magnitudes based on the results reported here.

### Codon Order Role in Sensitivity

It is well known that the position of codons has a significant role in elongation dynamics^43,44^. In order to study the relation between the order of codons and the sensitivity to perturbations, we induced mutations that result in the same or similar protein but changed the codon order (see Supplementary Methods S5, Fig. S3 and Table S1). We then compared the sensitivities of the original and the mutated gene. The analysis was done on the high protein abundance subset of 500 genes (defined below), as this group is expected to exhibit features related to the locations of codons (more details in the discussion section).

### Synthetic Gene Analysis

In some types of analysis, we discuss results obtained using a synthetic gene, mostly in order to control for various variables (which cannot be done in genomic data) and to gain some intuition. Thus, they may clarify, qualitatively, the expected behavior when real genes are used. A default synthetic gene is defined to have 500 codons (similar to the average ORF length in the discussed genome), and elongation rates *λ*_*i*_ = 1, ∀*i* ≥ 1.

### Experimental Data

Ribosomal density data was taken from^34^. A total number of 5,191 genes with ribosomal density measurements and sequencing data were analyzed in this research. About 3,600 of these genes have protein abundance measurements^45^. In some cases to be discussed later, a subset of 500 genes was selected (~10% of the discussed genome), representing protein abundance values; this set will be referred to as the ‘representative subset’. A subset of 500 genes with the highest protein abundance values will be referred to as the ‘high protein abundance subset’.

## Results

In all cases, unless otherwise stated, the results for negative perturbations (i.e. decreasing a considered rate/s) is presented. Positive perturbations resulted in a similar effect, but with a smaller magnitude and in the opposite direction, as expected (see Figs S4–S7). This will be further mentioned in the Discussion section.

We aim to provide useful estimation of *real* sensitivity values. In some of the results below, we provide numeric examples of an expected sensitivity change for a given change in the discussed parameter. The convention is that changes always refer to the absolute sensitivity. Both absolute and relative change examples will be shown. Note that if the studied parameters changes from *x*_1_ to *x*_2_, the relative change (in percentage) is defined as:

2 $$relative\,change\triangleq \frac{sensitivity({x}_{2})-sensitivity({x}_{1})}{sensitivity({x}_{1})}\times 100 \% .$$

For example, a certain increase in the initiation rate can result in absolute increase of 1% and relative increase  of 145% in sensitivity. Clearly, the relative change is only valid for the specific original values.

### The Sensitivity Profile Depends on Initiation Rate

**Figure 2:**
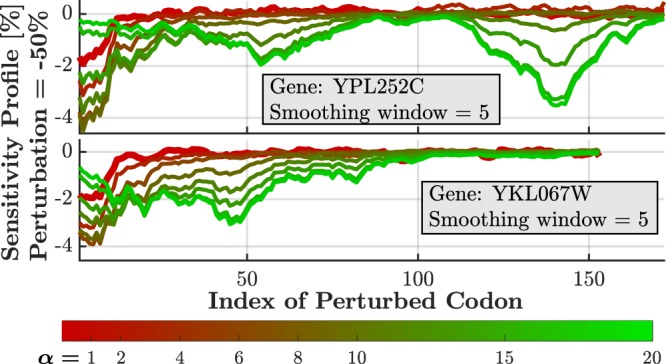
Sensitivity profiles of two specific genes for perturbation of −50% and different values of *α* (the factor at which the baseline initiation rate is multiplied). The genes YPL252C (Ferredoxin of the mitochondrial matrix) and YKL067W (Nucleoside diphosphate kinase) demonstrate how profiles change as *α* changes from the baseline value of 1 to 20. The profile continuously dependents on *α*, showing different characteristics for different values: Low-*α* profiles are sensitive at the first codons, while high-*α* profiles demonstrate sensitivity at the inner codons of the ORF. The profiles were smoothened by a moving average window of 5 codons. Few more examples are found in Fig. S8.

We first discuss the sensitivity profile for perturbed rate of single codons. In Fig. 2 we demonstrate the sensitivity profiles of two genes for a range of initiation rate factors *α*. We expect that as long as initiation is the rate-limiting factor of the system, internal regions will not be sensitive (i.e. perturbing the decoding rate in this region will not affect translation rate). On the other side, when other regions of the ORF are becoming the bottlenecks of the system, we expect them to be sensitive to perturbations. As the results below suggest, the first codons are of particular interest, showing relatively high sensitivity values. Moreover, the dependence of these values on initiation rate should be further investigated. Finally, these codons have an important role in controlling the flow of ribosomes.

**Figure 3:**
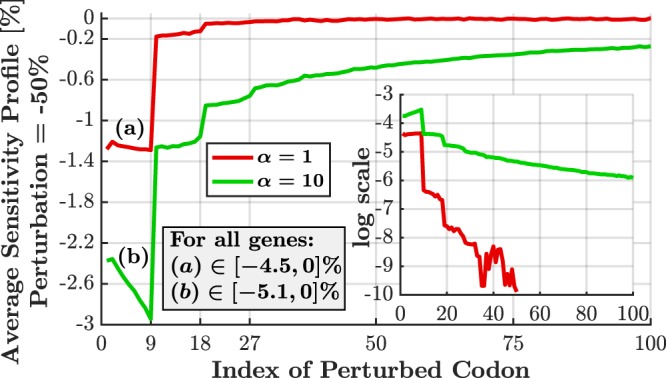
Average sensitivity profile for Yeast genome (5,195 genes) for perturbation of −50% and *α* = 1, 10 (*α* is the factor at which the baseline initiation rate is multiplied). First 100 codons are presented. High average sensitivity (~1.3% for α = 1, ~2.4–3.0% for α = 10) is observed in the first nine codons, along with a secondary weaker sensitivity (~0.2%, ~1.2%) region within the following nine codons. Although only the average values are shown, the range for all genes and for each *α* is indicated in the figure as (**a**) and (**b**). The α = 10 curve has gradually decreasing sensitivity with 0.1% at codon 192 (not shown in the figure). The subfigure shows the expected log-linear relation between sensitivity and codon location, as shown by Poker *et al*.^46^ for a mean-field approximation of TASEP. Due to extensive noise, the graph of α = 1 is shown only for i ≤ 50.

The average sensitivity profile of all *S. cerevisiae* genes, both for *α* = 1 and *α* = 10, is reported in Fig. 3 for the first 100 codons. For every codon along the ORF (indicated by its position index), the rate was changed from *λ*_*i*_ to 0.5*λ*_*i*_ at every one of the genes, one position at a time. All values per position were averaged resulting in the values presented in the figure. As can be seen, the first 9 codons are most sensitive, with decreasing (absolute) sensitivity at the following blocks of 9 codons. The steep transition at *i* = 10 can be explained by noting that reducing the elongation rate of any of the first 9 codons may directly affect the effective initiation rate because initiation is not possible as long as a ribosome is located (and delayed) in this region. This effect also exists in the following blocks of 9 codons, with decreasing magnitude.

It should be noted that the results generally agree with the analysis performed by Poker *et.al*.^46^: The authors analyzed a deterministic mean-field approximation of TASEP analytically and showed that in the considered scenario, the natural logarithm of sensitivity linearly decreases with the index *i*. In our case, as shown in the subfigure of Fig. 3, we see a similar trend, although the step-wise behavior was not reported in the deterministic analysis.

To summarize, observing sensitivity profiles reveals a non-trivial sensitivity behavior related to initiation rate. For baseline initiation rates, the first codons dominate the sensitivity to perturbations, emphasizing the critical importance in isolating these locations for proper analysis (as done in the following subsections), rather than simply taking the average sensitivity value of all codons. However, as further discussed below, for increased initiation rates, internal slower codons become the bottlenecks of the process and dominate the sensitivity.

### The Regimes of Sensitivity at the First Nine Codons

We now discuss the qualitative relation between the initiation rate and sensitivity. This subsection focuses on the first nine codons, which have been shown to have strong effect on the sensitivity. The next subsection considers the relations for the entire ORF.

We let $\xi ={\lambda }_{0}/({\langle {\lambda }_{i}\rangle }_{i=1}^{n})$, namely the ratio between initiation rate and the mean decoding rate. A single *ξ* value represents each gene. Such definition serves as a normalization of the initiation rate, which is essential in order to compare different genes.

**Figure 4:**
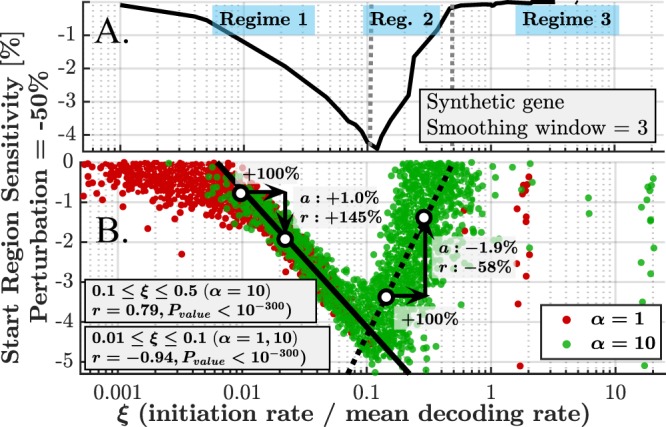
(**A**) Start region sensitivity (average sensitivity at the first nine codons) *vs*. *ξ* for a synthetic gene (for which *ξ* also equals to the initiation rate). There are 3 regimes for this relation: (1) sensitivity increasing with initiation rate (*ξ* ≤ 0.1); (2) sensitivity decreasing with initiation rate (0.1 ≤ *ξ* ≤ 0.5); (3) sensitivity is saturated and independent of initiation rate (*ξ* ≥ 0.5). These ranges are true for the discussed synthetic gene and may vary for other genes. (**B**) Start region sensitivity *vs. ξ* for *α* = 1 (red) and *α* = 10 (green). Each gene is represented by a single point for each *α*. In this figure, more isolated data points are represented by larger points for visual purposes. In the baseline case of *α* = 1, most of the genes occupy regime (1). When *α* = 10, many genes occupy both regimes (1) and (2). High Spearman correlations between start region sensitivity and *ξ* are obtained when considering regimes 1 and 2 separately (values are shown in the text-boxes). The data of *α* = 10 was used for linear estimation: when 0.01 ≤ *ξ* ≤ 0.1 a linear fitting yields $y=-3.4\times {\mathrm{log}}_{10}\xi -7.5 \%$, while for 0.1 ≤ *ξ* ≤ 0.5 we get $y=6.3\times {\mathrm{log}}_{10}\xi +1.9 \%$.

Figure 4A depicts the start region sensitivity versus *ξ* for the default synthetic gene (*λ*_*i*_ = 1). In order to understand this behavior, we define three regimes in terms of *ξ*. Regime 1: *ξ* ≤ 0.1. For extremely low *ξ*, the ribosomes are so sparse that very low sensitivity is observed (the distribution of the effective initiation times is very wide, so small variations in the start region cause no observable effective change). As *ξ* increases, the start region becomes more sensitive until the extremum sensitivity is achieved at *ξ* ≈ 0.1; Regime 2: 0.1 ≤ *ξ* ≤ 0.5. The sensitivity decreases with *ξ*, as now the system becomes less initiation rate limited due to slower codons along the ORF, reducing the sensitivity of the first nine codons. Regime 3: *ξ* ≥ 0.5. The first nine codons do not exhibit any sensitivity, even for increasingly large values of *ξ* (see Supplementary Results S6 and Figs S9–S11 for canonical examples with a synthetic gene).

Similar to the case of the synthetic gene, we can obtain start region sensitivity and *ξ* value for each of the *S. cerevisiae* genes. The results for *α* = 1 and *α* = 10 are shown in Fig. 4B, where each point represents a single gene. Based on the regimes described above, for baseline initiation rates (*α* = 1), most genes occupy regime 1. However, for *α* = 10, the genes occupy both regimes 1 and 2. Note than in general, the combined data resembles the behavior presented in Fig. 4A. Very few genes (for both *α* = 1 and *α* = 10) are found in the third regime, suggesting that such behavior is rarely observed in nature. It should also be noted that in contrary to the synthetic gene, many of the real genes at regime 3 have non-zero sensitivity. For these genes, a slow codon exists among the first codons, so increased *ξ* will not reduce the sensitivity of this region. High Spearman correlations between *ξ* and the perturbation level were found in both regimes 1 and 2 (values are shown in the figure). The results for *α* = 10 were used for linear fit and an estimation of change, which are presented in the figure.

These results also mean that our initiation rate estimation error of ±2% is valid, as it implies very small error in the sensitivity estimations (we demonstrated how a change of 100% in the initiation rate can lead to a −1.9% change in the absolute sensitivity value, thus an error of ±2% will result in a much lower expected change). The relation between start region sensitivity and a broader range of *α* values for the representative subset led to similar results, that are presented in Supplementary Results S7 and Fig. S12.

To summarize, we have shown, for the first time, that the sensitivity among the first codons is tightly related to a normalized initiation rate. Different regimes exhibit different behaviors in terms of sensitivity. Drastic change in the initiation rate may lead to “migration” of a gene to a different sensitivity regime, a mechanism that has not been discussed in literature so far.

### Various Sensitivity Profiles for High-***ξ*** Genes

Genes with high *ξ* values (i.e. ≥0.5) usually have sensitivity profiles that do not resemble the typical pattern in which the first nine codons are the only sensitive ones. Instead, these profiles take different forms as demonstrated in Supplementary Results S8 and Figs S13–S15. It turns out that for these genes the sensitivity of a codon is highly correlated to its decoding rate; for each of the 15 genes that satisfy *ξ* ≥ 0.5, the correlation between its sensitivities vector and decoding rates vector is between 0.42 and 0.74, with *p*_*value*_ < 1.5 × 10^−4^. Within genes that satisfy *ξ* < 0.5, less than 2% have significant (*p*_*value*_ < 0.01) correlation, and for this small sub-group of genes – the average correlation is 0.09.

For *α* = 10 there are much more profiles for which the sensitivity is dictated by the decoding rates of the internal codons, rather than the starting region. Roughly 39% of the genes exhibit significant (after Bonferroni correction with cutoff 1%) correlation (0.33–0.90) between TDR and the sensitivity of their codons.

### Higher Sensitivity for Shorter ORFs

**Figure 5:**
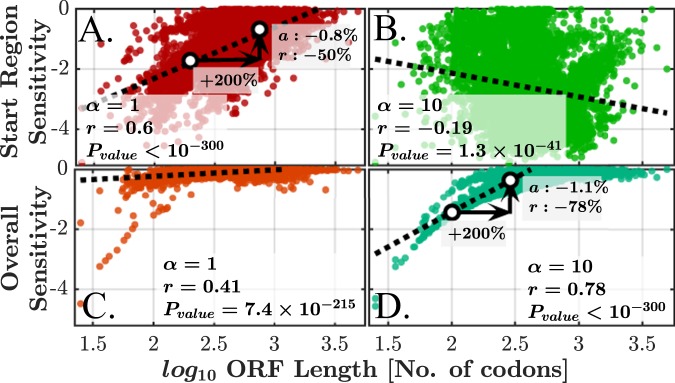
Start region sensitivity and overall sensitivity *vs*. ORF length for *α* = 1, 10, for a perturbation of −50%. Here *r* is the Spearman correlation, which was calculated in each scenario. (**A**) α = 1 and the first nine codons, linear fit yields ${\rm{y}}=1.7\times {\mathrm{log}}_{10}({\rm{length}})-5.7 \%$. (**B**) α = 10 and the first nine codons. (**C**) α = 1 and all codons are considered. (**D**) α = 10 and all codons, with a linear fit (only for ${\mathrm{log}}_{10}(\mathrm{length})\le 2.5$) is *y* = 2.3 × log_10_(length) − 6.0%.

The physical dimension of the ORF, i.e. its length in codons, may have a significant role in elongation dynamics. Here we analyze the connection between the ORF length and both start region sensitivity and overall sensitivity, for *α* = 1 and *α* = 10. This can be seen in Fig. 5. Indeed, when considering the first nine codons and *α* = 1 (Fig. 5A), shorter genes exhibit larger sensitivity in this region. Examining *α* = 10 reveals a different behavior: when considering the first nine codons (Fig. 5B), a negative correlation is observed. This is related to the fact that shorter genes usually have higher initiation rates (more details in Supplementary Results S9). Considering the overall sensitivity for *α* = 1 (Fig. 5C) and *α* = 10 (Fig. 5D), results in a significant positive Spearman correlation. Numeric examples appear in the figure.

Few other controls and aspects of the relation between the ORF length and the sensitivity were tested, such as controlling the elongation rate (measured by the mean of the typical decoding rate or MTDR^47^) and examining the relation between sensitivity at specific locations and length, all leading to similar conclusions (details in Supplementary Results S9 and Figs S16–S19).

### Sensitivity and Protein Abundance

The corresponding protein abundance (PA) is an important property of a given gene. Thus, it is desirable to study the relation between PA and the sensitivity profile of a gene. We focused on the relation between PA and the start region sensitivity for *α* = 1 and between PA and the overall sensitivity for *α* = 10.

**Figure 6:**
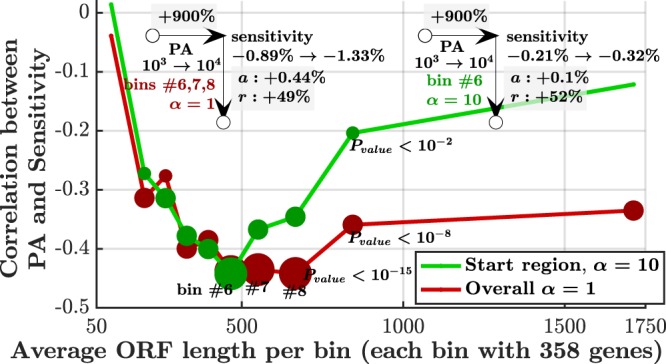
Correlation between PA (protein abundance) and sensitivity. Genes are divided into 10 bins of genes with similar ORF length. Each bin is represented by the mean length of the 358 genes it contains. More significant results are represented by larger dots (3 sizes for 3 thresholds that are marked in the figure). For *α* = 1 (red) only the first nine codons are considered (start region sensitivity). For *α* = 10, the sensitivity is averaged across all codons. In both cases, the most significant bins (6, 7, 8 for *α* = 1 and 6 for *α* = 10) were used to estimate the expected sensitivity change, which is presented in the figure for PA changing from 10^3^ to 10^4^; for example, in the case of *α* = 10, when PA changes from 10^3^ to 10^4^ (which is a 900% change), the start region sensitivity changes from −0.89% to −1.33%, which is an absolute change of 0.44% or relative change of 49%.

We showed that the sensitivity is correlated with the ORF length and it is known that this length is correlated with PA^47^. Thus, in order to control for this dependence, we examine the correlation between PA and sensitivity for sub-groups of genes with similar ORF lengths, divided into 10 bins with equal number of genes, as presented in Fig. 6. Interestingly, for *α* = 1, highest correlations were achieved for genes with 400–700 codons. This may be related indirectly to the specific functions and evolutionary constraints related to genes in these bins. In the case of *α* = 10, correlations were slightly lower for most of these lengths. More aspects of this relation, such as MTDR control due to correlation with PA^48^, can be found in Supplementary Results S10 and Figs S20–S24.

### Perturbation Magnitude

**Figure 7:**
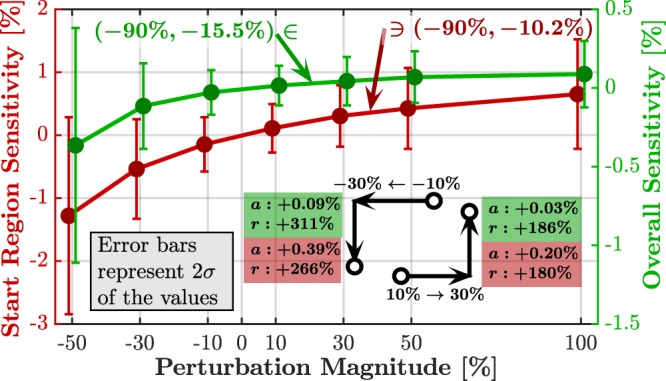
Sensitivity *vs*. perturbation magnitude. As before, for *α* = 1 only the start region sensitivity was considered (first nine codons; red plot in the graph), while for *α* = 10 the overall sensitivity was considered (all the codons were used for averaging; green plot in the graph). Labels show sensitivity values for perturbation of −90%, which reveal the very-steep relation between sensitivity and perturbation magnitude. A numerical example shows the sensitivity change for perturbation increasing from 10% to 30% both for positive and negative case.

The chosen magnitude of ±50% for the studied perturbations was somewhat arbitrary. In order to test how sensitivity is affected by different perturbation magnitudes, the representative subset was subjected to several values of perturbations: {−90%, +100%, ±50%, ±30%, ±10%}. For each perturbation value, start region sensitivity was calculated for *α* = 1 and overall sensitivity was calculated for *α* = 10, as presented in Fig. 7. As expected, the effect is stronger for negative perturbations (further explained in the Discussion section). These results can serve as a useful guideline for estimating sensitivity for various perturbation magnitudes by means of simple linear interpolation.

### Prolonged Perturbations

**Figure 8:**
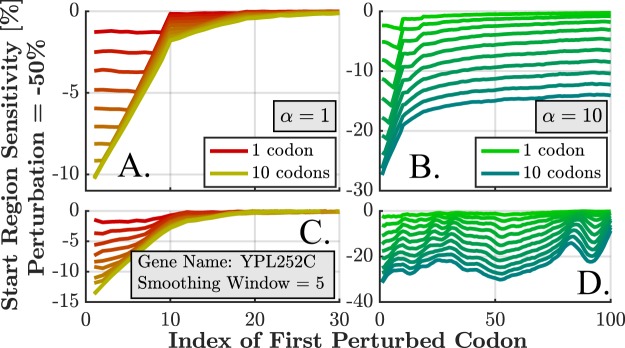
Sensitivity profiles for prolonged perturbations, from 1 up to 10 codons long (with corresponding 10 plots at each of the subfigures). Each index along the horizontal axis represents the first location of the perturbation. For *α* = 1 the sensitivity at the first *s* + 1 − *L*_*p*_ codons can be estimated as *y* = −0.3% − 1.1*L*_*p*_ (see Supplementary Results S11). (**A**) The average profiles of the representative subset of genes for *α* = 1. (**B**) Similarly for *α* = 10. (**C**) The profiles of a specific gene YPL252C for *α* = 1. (**D**) Similarly for *α* = 10.

So far, we have discussed localized perturbations that are associated with a specific codon and location. In reality, the disturbance can take place at several adjacent locations (for example, due to a local mRNA fold or miRNA binding that affects the elongation time at a region of codons). Figure 8 shows the results for both *α* = 1 and *α* = 10, for the representative subset of 500 genes and different perturbation lengths *L*_*p*_ (1 to 10 codons). Figure 8A,B show the average profile for all discussed genes, while Fig. 8C,D show the profiles of an example gene (YPL252C) for the corresponding values of *α*. For *α* = 1, the sensitive region is located at the first (*s* + 1 − *L*_*p*_) codons (recall that *s* is the ribosome size in codons), with sensitivity that is linear to *L*_*p*_. For *α* = 10 this relation is linear only for high *L*_*p*_ values and is location dependent. More details found in Supplementary Results S11 and Fig. S25.

### Elongation-Related Factors Perturbation

**Figure 9:**
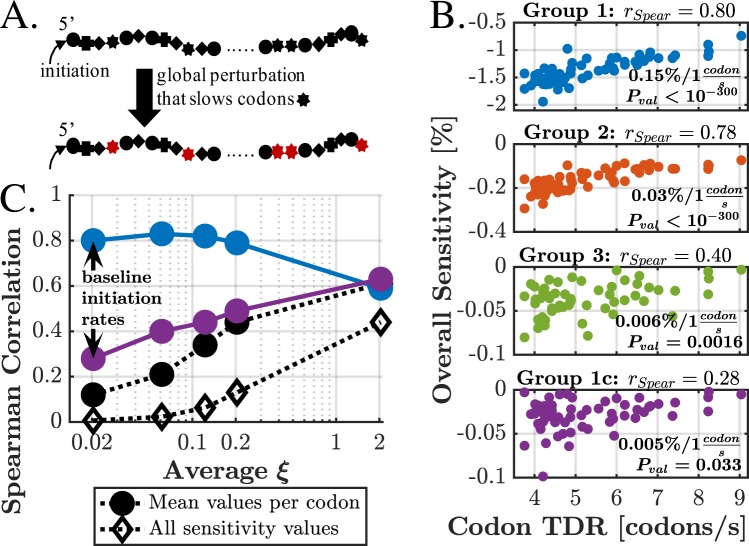
(**A**) Illustration of the procedure of perturbing codon globally. Each shape represents a certain type of codon. We induce a perturbation that is associated with the ‘star shaped’ codons, which now become slower (for example) along the entire ORF. (**B**) Overall sensitivity *vs*. decoding rate, for four different sub-groups, defined in the main text. Each subfigure corresponds to such sub-group and includes the estimated sensitivity change for a given change in decoding rate. (**C**) Spearman correlation values of overall sensitivity *vs*. elongation rate for different *ξ* values. Group 1, which is associated with first 9 codons, indeed exhibits lower correlation for higher initiation rates, as expected. Other correlations increase. “All sensitivity values” is the group of all 60 × 5,191 data points, while “Mean values per codon” is a group of 60 values, averaging all relevant genes per codon.

Here we consider a global type of perturbations, which affects the availability of specific tRNA type. This availability is affected by the presence of amino acids, enzymes (e.g. aminoacyl tRNA synthetase), specific transcription factors that regulate translation, etc. To model such phenomena, we induced a change in the elongation rate of a given codon across the entire ORF and observed the change in the translation rate (see Fig. 9A). To avoid a bias, we ignored perturbations in the start codon ATG (Methionine).

Allegedly perturbing slow codons (e.g. by slowing them) should have a greater effect on translation rate than perturbing fast ones. This conjecture, however, greatly depends on the initiation rate. We first consider the case with the baseline initiation rate values. Then, we discuss how the conclusions change when *α* (thus 〈*ξ*〉) increases.

For *α* = 1, no significant correlation between sensitivity and TDR was found. We conclude that the decoding rates of the codons by themselves cannot explain the average sensitivity; in order to address this issue, we must consider additional factors which “blur” this relation and are described below.

Knowing (from previous sections) that the start region may have an important role in determining the sensitivity, we would like to distinguish between ORFs that contain a perturbed codon along the start region and ORFs that don’t. We maintain this distinction by introducing the following definitions of gene sub-groups. An ORF of a gene *G* is defined as a vector of codons (*c*_1_, …, *c*_*n*_). For example, *c*_1_ = *AUG* for most genes. We define a group of genes *Γ* for each codon type *C* as follows: *G* ∈ *Γ*(*C*; *I*_*yes*_, *I*_*no*_) if and only if $C\in {\{{c}_{i}\}}_{i\in {I}_{yes}}$ and $C\notin {\{{c}_{i}\}}_{i\in {I}_{no}}$, where *I*_*yes*_ and *I*_*no*_ are groups of codon indexes. In other words, a gene will belong to the group if and only if it has codon *C* among the *I*_*yes*_ codons set, but not among the *I*_*no*_ codons set. We next define four groups: Group 1 is *Γ*_1_(*C*) = *Γ*(*C*; {2, …, 9}, Ø) (we exclude the start codon). This means that *Γ*_1_(*CGA*), for example, contains all genes that contain *CGA* at least once within codons 2–9. Specifically, for *Γ*_1_(*CGA*), the group of codons that must not contain *CGA* is the empty group. Group 2 is *Γ*_2_(*C*) = *Γ*(*C*; {10, …, 18}, {2, …, 9}) and group 3 is *Γ*_3_(*C*) = *Γ*(*C*; {19, …, 27}, {2, …, 18}). So far, we have 3 groups for each one of the 60 codons (a total of 180 groups); 50% of these groups have at least 530 genes, and no group has less than 85 genes. Finally, group 1c (1-complimentary) is *Γ*_1*c*_(*C*) = *Γ*(*C*; {10, …, *n*}, {2, …, 9}), where *n* represents the last location of every discussed gene. Each “group 1c” has thousands of genes. For each codon *C* and each group, the average sensitivity value was calculated and presented versus the typical decoding rate of *C*. These results are presented in Fig. 9B. More control methods are discussed in Supplementary Results S12 and Figs S26–S28.

We now consider the case of increased initiation rate. We observe that as the mRNAs become less initiation rate limited, the importance of the first codons is decreased, giving rise to correlation between overall sensitivity and the decoding rate of the perturbed codon, as can be seen in Fig. 9C. We see that as *ξ* increases, the correlation in group 1 is decreased, while group 1c (which represents all other genes per codon type) exhibits increasing correlation, implying that the relation between elongation rate and sensitivity is now observed across all downstream codons, rather than only the first few. Finally, when ignoring position control and discussing all codon indexes (whether if averaging the sensitivities per codon type or not), the discussed correlation increases with *ξ*.

To summarize, the somewhat-complicated method of control turned out to be necessary. When perturbing the availability of a given codon type, and for a low initiation rate, the overall effect on protein synthesis greatly depends on the presence of this codon along the start region of the ORF. Such analysis further emphasizes the importance of identifying the regime in which the mRNA operates, in order to gain full understating regarding perturbation susceptibility.

### The Effect of the Codon Order on the Sensitivity

Below we present the comparative results of the real genome to the first of two types of mutations, as briefly defined in the Methods section and in Supplementary Methods S5. In the first type, the mutated genes are preserving the biological properties of the gene, but with altered codon order, eliminating any effect related solely to codon order. The second type (in which the amino acids are not strictly preserved but rather allowed to be changed into similar ones) led to similar results and presented in Supplementary Figs S29–S31. For both types and for each of the 500 analyzed genes, 10 mutative variants were generated. We tested whether the average sensitivity of the original gene in a given region of the ORF is significantly different than the corresponding average sensitivity of the mutative genes at the same region. Let us observe a region starting at codon *a* and ending at codon *b*. For each gene *g* we let *O*_*g*_ to be the average (from codon *a* to *b*) original sensitivity, i.e.:

3 $${O}_{g}\triangleq {\langle S{P}_{g}^{orig}(i,\,p,\,\alpha )\rangle }_{i=a}^{b}=\frac{1}{b-a}\,\sum _{i=a}^{b}\,S{P}_{g}^{orig}\,(i,\,p,\,\alpha ).$$

Now, denote by $S{P}_{g,\,k}^{mut}$ the sensitivity profile of the *k*^*th*^ mutation variant of the gene *g*, which is one of 10 randomizations of codon-order that preserve protein composition and the codons frequencies in the ORF (i.e. codon usage bias), but eliminate any location-specific effects (e.g. slow codons at the 5′ end of the ORF^49^). For each gene *g* we have:

4 $${M}_{g}\triangleq {\langle {\langle S{P}_{g,\,k}^{mut}(i,\,p,\,\alpha )\rangle }_{k=1}^{10}\rangle }_{i=a}^{b}=\frac{1}{10(b-a)}\sum _{k=1}^{10}\,\sum _{i=a}^{b}\,S{P}_{g,\,k}^{mut}\,(i,\,p,\,\alpha )$$

(10 mutative variants are averaged to get a single mutative profile, which is further averaged from *a* to *b*). The set *Δ* = {*O*_*g*_ − *M*_*g*_} is then hypothesized to have a mean of zero and is tested with t-test. For all *a* ≤ *b* ≤ 200 we calculated the $-\,{\mathrm{log}}_{10}\,({p}_{value})$ of the test, and denoted it by *Π*_*a*,*b*_. The significance threshold is defined by Bonferroni correction with a cutoff of 1%, which is ~4 × 10^−7^ (i.e. *Π*_*a*,*b*_ ≥ 6.4).

**Figure 10:**
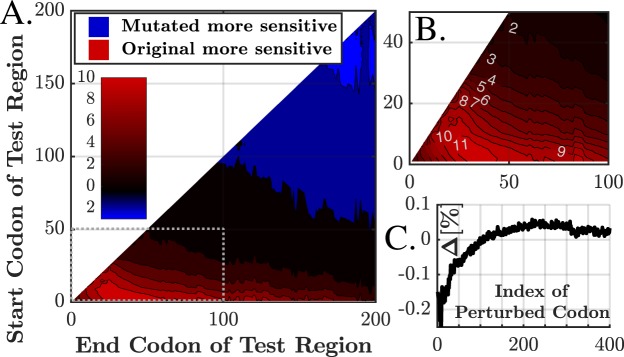
(**A**) *Π*_a,b_ values, representing the significance of sensitivity difference between original and mutative genes, e.g. a value of $-\,{\mathrm{log}}_{10}(0.01)=2$ represents a 1% significance (in either direction). Here *p* = −50%, *α* = 10. The color indicates the direction of change: red (blue) represent that the original (mutated) values were more sensitive, with *P*_*val*_ of up to 10^−10^ (10^−2^). (**B**) sub-region of interest with significant change. The region of codons 5–100 (roughly) exhibits statistically significantly higher absolute sensitivity in original genes than in mutative genes. For example, consider start codon (y-axis) of 10 and end codon (x-axis) of 30. The *Π*_10,30_ value is ~10, which in this case means that the original region of codons 10–30 is more sensitive than the mutated region of same codons, with *P*_*value*_ = 10^−10^. (**C**) The difference between average original and mutative profiles. First 400 codons are shown, revealing regions with positive or negative change. For the previously discussed region of codon 20–50, the difference is 0.07–0.15% (depends on the exact location).

Figure 10A shows *Π*_*a*,*b*_ for *α* = 10 (*α* = 1 led to no significant effect), with Fig. 10B showing a smaller sub-region of interest. Figure 10C depicts the difference between the average original and mutative profiles, namely 〈*O*_*g*_−*M*_*g*_〉, averaging all discussed genes (the exact profiles and further details can be found in Supplementary Results S12). These results may be explained by the presence of relatively slower 30–50 first codons^49^; due to slower decoding rates, this region also tends to be more sensitive. Mutations eliminate the slower region, reducing the absolute sensitivity. On the other hand, these codons are now distributed along other regions of the ORF, leading to slightly increased sensitivity in the mutant variants.

To summarize, we tested whether the slow 30–50 codons of the 5′ end of the ORF are related to sensitivity, by creating random variants that eliminate such phenomena but preserve the biological meaning of the sequence. It turns out that indeed, roughly the first 100 codons tend to be more sensitive, while codons 100–200 tend to be less sensitive.

### Functional Analysis

In order to examine the relation between the sensitivity and the function of a gene, we focused on perturbations that are originated in global cellular processes, resulting in a decoding rate change across all codons of a given type (similarly to section “Elongation-Related Factors Perturbation”). For each gene, we calculated the average sensitivity of all unique codons that appear in the ORF, resulting in a single value per gene. We then classified the genes into sensitive and insensitive genes with different percentiles and performed gene ontology analysis using^50^. Another approach was grouping the genes based on slim-GO terms and comparing the sensitivity of each group with the background. Interestingly, among others we found that gene groups related to various aspects of translation regulation to be more sensitive than other genes; this may be a feature directly selected for in highly expressed housekeeping genes. All details, as well as another functional analysis based on location specific perturbations, are found in Supplementary Results S13 and Figs S32–S35.

## Discussion

We have simulated and quantified, for the first time, the effect of several mechanisms of translation perturbations on the translation rate, i.e. translation efficiency of *S. cerevisiae* genes. The sensitivity profiles were characterized by three regimes of the relation between sensitivity and *ξ*, the ratio between initiation rate and mean elongation rate (Fig. 4). Analysis was performed both for the baseline initiation rates (*α* = 1) and for the 10-fold increased ones (*α* = 10), revealing the full space of possible regimes in real genes. Each analysis revealed an additional conclusion that depends on both *α* and the discussed regime. The first nine codons (the typical size of the ribosome) are particularly important as they exhibit increased sensitivity (up to 5%) for the vast majority of genes (Fig. 3). The sensitivity in this region is highly correlated (up to 0.94) with *ξ*. Such high correlation implies that *ξ* can serve as a very good sensitivity predictor, given initiation and elongation rates. The sensitivity is highly correlated (up to 0.78) with ORF length (Fig. 5) in several scenarios and with protein abundance (up to 0.43 for some lengths, Fig. 6). In cases where *ξ* ≥ 0.5, the sensitivity profile is dominated by the local codon elongation rates rather than by the initiation rate or *ξ*. These results imply that the initiation rate is a key factor in determining the sensitivity profile. Moreover, we have gained tools to qualitatively estimate the expected profile. Along with single-codon perturbations, we have analyzed prolonged perturbations. As expected, these have resulted in higher sensitivities which are almost linear with the perturbation length (Fig. 8). For example, perturbing the rate of block of 10 codons by 50% can have an effect of up to 35% on the translation rate.

The reported results were focusing on negative perturbations (lowering the decoding rates). In all cases, the results for positive perturbations to the same extent resulted in similar conclusions but with a lesser sensitivity magnitude. This is clear considering the fact that translation rate is usually limited by rate bottlenecks (whether in the initiation process or somewhere along the ORF). Thus, reducing rates might create new bottlenecks or strengthen existing ones, while increasing rates, unless tailored specifically at the bottlenecks, will result in a smaller changes, which we quantified and reported (Fig. 7).

We have emphasized the importance of the first codons in the context of perturbations sensitivity. It is also known that the first codons may have a role in controlling the initiation rate^49^ (e.g. the first codon after AUG, which is part of the Kozak consensus sequence^51,52^), and various additional phenomena related to gene expression. However, researchers currently believe that most of the signal is related to the 5′ UTR and the scanning mechanism of the pre-initiation complex^53^. Our method of initiation rate estimation effectively includes all such mechanisms, resulting in predicted ribosomal density that is similar to what was reported empirically.

It is important to emphasize that the reported sensitivity among the first codons is not related to the initiation itself, but rather to relationship between the total initiation rate and the further elongation step, which we described as sensitivity regimes. This can be easily seen since: (1) the phenomena we studied here do not affect the composition of nucleotides at the coding region; thus, we do not expect any variation in the assembly of the pre-initiation complex. All discussed phenomena in this paper are external and relate to changes in resources (e.g. tRNA levels), which are required for the translation elongation steps. (2) According to the canonical model of translation in eukaryotes, the nucleotides related to the recognition of the start codon by the pre-initiation complex (i.e. the Kozak sequence^52^ mentioned above) only refer to the first codon after the start codon, while we showed that the sensitivity is increased at the first ~9 codons.

Global elongation-related factors perturbations were also simulated and reported. This analysis further emphasizes the importance of the first codons in determining sensitivity. When a proper control was performed, a correlation of 0.8 was found between the sensitivity and elongation rate. This relation was masked if all the ORF codons were considered, without isolating the first codons, which were the main source of sensitivity when the initiation rate is low. However, for increased initiation rates, the importance of the first codons decreases, while the discussed correlation appears along all regions and codons (Fig. 9).

The results reported here emphasize the region of 9–18 codons at the beginning of the coding region; this region overlaps with many additional signals related to the translation process that have been reported in the recent years^49^; and the results agree with recent study that also emphasized the effect that this region has on translation^38^. Specifically, our results suggest that evolution may shape the codon composition in this region also in the light of their important effect on translation changes due to perturbations in elongation-related factors.

We believe that the reported results are robust and provide a good framework of guidelines for addressing perturbations both in synthetic and biological systems. The fact that the qualitative behavior was not Yeast-specific, but was also observed in synthetic genes, suggests that the general trends observed here will appear also in other model organisms. We have shown that the main source of sensitivity is located in the beginning of the ORF, making it an important subject for discussion when addressing the sensitivity of mRNA translation.

It is also important to mention some limitations of the model. First, we assumed that the global cell dynamics are slow enough compared to the translation elongation process, so constant perturbations are a good approximation. There may exist shorter spikes in translation that are not analyzed here; however, these may have smaller effect due to their short period. In addition, the elongation rates in our analysis were based on typical decoding rates (after filtering biases and positions with extremely low decoding rates; see the Methods section). Thus, the results reported here under-estimate the effect of the elongation perturbation on translation. We also did not assume that the initiation is affected by elongation due to a finite pool of ribosomes^29^. Again, this is expected to cause an under-estimation of the elongation perturbation effect on translation. All these aspects should be further considered in the future. It should also be noted that even though we used a fixed value of *s* = 9 for the ribosome size, it may change due to different conformations in different stages^54^. However, our results seem to be invariant to small changes in this parameter.

Finally, the reported results can be further validated experimentally in the future. The easiest system to start with is cell free translation system^55^, where concentrations of translation-related factors can be modulated relatively easily. In order to create a rate reduction at specific location, a region under consideration can be modified by inserting a complementary mRNA oligo (or RNAi), serving as a ribosome slow-down mechanism that results in effective average rate elongation reduction in this region.

To summarize, this work sheds light on some non-trivial aspects of sensitivity to perturbation in the translation process. We have provided a thorough analysis for various cases, resulting in typical values with biological meaning and a framework for a proper control and analysis of similar questions.

## Electronic supplementary material

Supplementary results and methods

dataset 1

dataset 2

dataset 3
