# Supplementary material for: Genome-Scale Analysis of Perturbations in Translation Elongation Based on a Computational Model
Source: Sci Rep. 2018 Nov 1;8:16191. doi: 10.1038/s41598-018-34496-3 (PMC6212587; doi:10.1038/s41598-018-34496-3)
Supplement: Supplementary file 1 — Supplementary results and methods [file 41598_2018_34496_MOESM1_ESM.pdf]

Supplementary Information

Genome-Scale Analysis of Perturbations in Translation Elongation Based on a Computational Model

Doron Levin and Tamir Tuller

Supplementary Methods

S1. Detailed description of TASEP implementation

A 1-dimensional array of the form $O = (I_1, I_2, \dots, I_n) \mid I_i \in \{0,1\}$ represents ribosome location on the discussed ORF, which has a length of n codons. A vector of elongation rates is given as $\underline{\lambda} = (\lambda_1, \lambda_2, \dots, \lambda_n)$. We define initiation as a transition of the ribosome from a virtual location $i = 0$ to the first real codon $i = 1$. The transition time from location i to $i + 1$ is distributed exponentially with mean λ_i^{-1}, where λ_0 denotes the initiation rate. Note that the effective initiation rate is usually lower due to unsuccessful initiation attempts (e.g. when the first ribosome is too close and does not allow a new one to assemble).

At every iteration, two steps are performed:

1. The "next jumper" (the ribosome which is the next to attempt a transition from its site to the next one) is identified, so that the chance of a ribosome at location i to be chosen is proportional to λ_i^{-1};
2. The action (transition of waiting) of the jumper is determined: if the next (downstream) ribosome does not limit the movement, a transition can take place. Otherwise – it remains at the same place. If the ribosome was located on the last codon, it will terminate, symbolizing a newly created protein.

It should be mentioned that in order to efficiently identify the next jumper, a property of exponential distributions was used: the minimum of exponentially distributed random variables is also exponentially distributed.

In this kind of simulations, large number of iterations must be performed in order to reach steady state. We first iterate until the system reaches steady state (step 1), then begin to calculate mean translation rate, which is the no. of terminations (i.e. no. of proteins produced) averaged over time (step 2). For our purpose, steady state was defined as the point in time where the termination process becomes stationary. 10^7 iterations were used in all cases. Next, the number of iterations in steady state had to be determined. In order to guarantee similar errors in all kinds of mRNAs, the iterations continued until a change smaller than 1% in termination rate was observed 10^6 iterations apart. However, due to the discrete nature of the process, the convergence might be very gradual (discrete changes leave "long term" marks on the average). To overcome this issue, the process described above was performed several times and the results averaged, allowing us to reduce the statistical noise. Specifically, the reference case (without perturbations) was performed 100 times, while perturbed simulations were performed 20 times. Note that the stochastic simulation noise was a major difficulty in obtaining viable results in our analysis.

S2. Sensitivity profiles for $\alpha = 1$ and different values of ribosome size s

The sensitive region near the initiation site is tightly related to the ribosome size s, which is a model parameter. However, this parameter does represent the biophysical dimension of the ribosome, as indicated in the main text. We do not expect a change in the sensitivity in the region when s is changed (e.g. due to different conformations), as long as $s \ll n$, when n is

the length of the ORF in codons. The profiles in Figure S1 are generated based on simulation of ~ 500 representative genes with $s = 11$ and $s = 13$.

These results are not surprising, as the (absolute) sensitivity increases at the first codons due to an increase at the time that the first ribosome is limiting initiation, which is true as long as it is located in the first s codons. Thus, although rigorous proof is not provided, we expect the results presented in the main text to be invariant to the actual conformation of the ribosome, up to small changes like the exact position of the transition of sensitivity (which is expected to occur after s codons, as demonstrated in Figure S1).

S3. <u>Initiation rate estimation</u>

Suppose that the *unknown* effective initiation rate for a specific gene is λ_0 (see note (*) below). For this gene, we have elongation rates $\lambda_1, \dots, \lambda_n$ when n is the number of codons in the ORF of the gene. Given these parameters we can simulate the elongation dynamics until steady state is reached, as described in supplementary methods S1. Let's denote by $\langle N_R \rangle = f(\lambda_0, \lambda_1, \dots \lambda_n)$ the average number of ribosomes on the mRNA in steady state (see note (**) below). We also denote by N_R^{data} the experimental number of ribosomes, from Arava *et al*[1]. It is very probable that these quantities represent a steady state elongation process, as reaching steady state takes much less time than a typical active elongation process.

We require that the initiation rate will satisfy: $\hat{\lambda}_0 = \mathrm{argmin}_{\lambda_0} \left| \langle N_R \rangle - N_R^{data} \right|$. However, as there is no analytical solution for the equation above, the estimation had to be done numerically for each of the ~5000 considered genes.

We used the following 2-step approach:

1. Find the number of iterations needed to reach steady state;
2. Using the number of iterations, perform binary search (see note (***) below) to minimize $\left| \langle N_R \rangle - N_R^{data} \right|$ by altering λ_0.

The first step is required for computational efficiency: the number of codons across all genes changes between several dozens and several thousands. Using a number of the same number of iterations for all genes (which has to be large, to reach steady state at the longest genes), will results in very long unnecessary computational time.

Let's denote by $\{N_R\}_i^k$ a group of k values of number of ribosomes, achieved from k simulations with i iterations each. To complete the first step, the number of iterations i was increased until it was impossible the reject the hypothesis that $\{N_R\}_i^{10}$ and $\{N_R\}_{3i}^{10}$ were resulted from the same distribution (using Kolmogorov-Smirnov test with 1% confidence). Then i was increased again by a factor of 10: $i_{steady} = 10i$.

Now, the second step can begin. The binary search was performed by defining $\lambda_0 = 10^p$ when $p \in [-3.5, 2.5]$. At each iteration of the binary search, 100 simulations were performed with i_{steady} TASEP iterations, to receive a good estimation of $\langle N_R \rangle$. The binary search stopped when $\frac{\left|\log\langle N_R \rangle - \log N_R^{data}\right|}{\left|\log N_R^{data}\right|} < 0.5\%$ (in some cases the threshold was relaxed to 1%).

After achieving $\hat{\lambda}_0$ values for all the genes, an error estimation as performed. For every gene, 100 simulations were done with 10^6 iterations to reach steady state and 10^6 more in steady state. A value of $\langle N_R \rangle$ was achieved and estimation error was calculated for each gene by $e = \frac{N_R^{data} - \langle N_R \rangle}{N_R^{data}}$. An empirical CDF is shown in Figure S2, where $\hat{\mu} = -0.02\%$ (std. error of 0.01%) and $\hat{\sigma} = 0.75\%$ (std. error of 0.01%).

Note that this is an error in the estimated number of ribosomes. However, it is known that the relation between the initiation rate and the number of ribosomes is linear (with a slope of 1) when the system is initiation rate limited (i.e. in the low density flow phase of TASEP), and the slope decreases for higher initiation rates until complete saturation. This means that the error range above represents the error of the initiation rate itself in the initiation rate limiting regime, where that error matters the most.

Note (*): In this context, we refer to initiation in the narrow sense of 'decoding' the start codon, ignoring the previous steps. This simplification is valid as we incorporate the duration of these steps in the effective initiation time.

Note (**): Here, in contrary to the main text and supplementary methods S1, the steady state was defined from the standpoint of number of ribosomes, which, in general, may differ from termination rate steady. However, in practice, we do not expect these definitions to be significantly different because the termination rate is tightly related to the ribosomal occupancy.

Note (***): Binary search here was utilized in the classical iterative sense: wide boundaries for initiation rate scan were defined. At each iteration, the value at the middle of the range was tested and new range was defined according to the result. This is done until the range is narrow enough to specify an accurate initiation rate value.

S4. The bottleneck factor

We can define a 'bottleneck factor' to indicate how much the system is initiation limited. One must consider the physical size of the ribosome and account for the fact that s codons must be translated before an additional initiation can occur. Thus, we may define the bottleneck factor ξ_{BN} as the ratio between initiation rate and the minimum *effective* elongation rate of any s codons in the ORF. In other words, for a give gene we define:

$$\xi_{BN} = {}^{\lambda_0}\!\big/_{\min\left[\lambda_{eff}(i, i+s-1)\right]}, \qquad 1 \le i \le n-s+1$$

where $\lambda_{eff}(a,b) = \left(\sum_{i=a}^{b} \lambda_i^{-1}\right)^{-1}$. When discussing the baseline initiation rates, the average bottleneck factor across all discussed genes is $\langle \xi_{BN} \rangle = 0.22$, demonstrating the importance of elongation in the translation process. The factor ξ, which is defined in the main text, serves as a similar indicator. It is less bio-physically accurate, but more intuitive and easier to calculate. Moreover, the predictive quality of ξ_{BN} was not better than the predictive quality of ξ.

S5. Mutations for Testing Relation between Codon Order and Sensitivity

We suggest 2 types of mutations, while preserving the codon usage bias:

(1) Synonymous mutations that fully preserve the amino-acid composition of the protein;
(2) Mutations inside a cluster of similar amino-acids (defined below), resulting in a composition that is similar to the original one.

On both cases, 10 mutative variants were generated for each discussed gene.

The first suggested mutation type is simply changing the original codons to synonymous ones (so that the protein composition remains unchanged) while preserving the codon usage bias.

The second type introduces somewhat more "aggressive" permutations, and is performed by 3 steps:

1. We group all amino-acids into clusters by a method to be described below.
2. Codons in each group (cluster) are shuffled.
3. Each codon is now changes to synonymous one while preserving codon usage bias (similarly to first type mutation).

The clustering is done hierarchically using Grantham's distance[2] metric, which is based on 3 factors to measure similarity between amino acids: composition, polarity and molecular volume. These distances were inserted to a hierarchal clustering algorithm, resulting in the dendrogram shown in Figure S3.

The numbers are associated with the amino acids as described Table S1 (see column "#"). The cutoff was chosen at 8 clusters.

Supplementary Results

S6. Synthetic (default) gene examples

An important canonical example regarding the sensitivity of translation rate is a uniform elongation rate gene with initiation rate that is equal to the elongation rate. A length of 500 codons was used. In Figure S9 we can see the sensitivity profile for negative and positive perturbations.

Observing the sensitivity profile for negative perturbations, we see that the sensitivity is low at the edges, with maximal absolute values at center of the gene. This can be explained qualitatively as follows: When the system is not initiation rate limited, we have a stationary flow of ribosomes without any bottlenecks, thus sensitivity only depends upon interaction between ribosomes; we now claim that the change in the translation rate (when a perturbation is introduced) must result from combined change in both initiation and termination ends. However, if the effect exists only on one end, it is masked by the stochastic flow from or to the other end. If, on the other hand, the effect exists at the center, the "masking region" is 2-times smaller, thus an observable effect appears.

	**Effect on initiation end**	**Effect on termination end**	**Combined effect**
**First ribosomes**	strong	negligible	negligible

**Central ribosomes**	moderate	moderate	moderate
**Last ribosomes**	negligible	strong	negligible

A synthetic gene simulation was performed to test the dependence on initiation rate. Figure S10 demonstrates $SP(i, -50\%, \alpha)$ for the first 50 codons for a range of α values which satisfy $\lambda_0' \leq 1$ (the parameters of the genes are described in the figure). There is also a sharp transition at $i = 10$, similar to what was observed for real genes in the main text. This can be easily explained by noting that reducing the decoding rate of any of the first nine codons, directly affects the *effective* initiation rate because initiation is not possible as long as a ribosome is located (and delayed) in this region. The effect is expected to exist in the following nine codons as well, but with a significantly lower magnitude.

Finally, we show the regimes graph for synthetic gene with positive perturbations in Figure S11.

S7. Sensitivity at first 9 codons for wide range of α values

We have extensively discussed the relation between sensitivity and initiation rate. We used the representative subset to examine the start region sensitivity versus different factors of initiation rate, from 0.1 up to 10, while the factor '1' represents the baseline initiation value. The results are shown in Figure S12. We can roughly estimate that if the initiation rate is increased by 10% (relative to the baseline), the average sensitivity will be absolutely increased by 0.11% (or relatively by 8%), while if we decrease it by 10% the sensitivity will be absolutely decreased by 0.07% (or relatively by 5%).

These results also partially show the behavior of the regimes. When the initiation rate is decreased enough, the average sensitivity become negligible (regime 1). If the initiation rate would have been increased enough, we would see a reduction in the sensitivity again (probably not to zero, but some other saturated value), due to another reason (regime 3), as has been described in the main text.

S8. Profiles of genes with high ξ and relation to TDR

In Figure S13 we see the sensitivity profiles of 15 genes that satisfy $\xi \geq 0.5$ for the baseline initiation rates. In Figure S14 we show the sensitivity versus the typical decoding rate (each point is a single codon at a single gene) for each of these 15 discussed genes, with colors corresponding to Figure S13. Clearly, for $\alpha = 10$ we expect much more profiles to behave similarly and exhibit correlation between the sensitivity and the TDR of a codon. Indeed, as demonstrated in Figure S15, roughly 37% percent of the genes show significant correlation between the sensitivity and the TDR of their codons. The figure shows the P_{value} and the correlation between the sensitivity and TDR for each one of the 5,191 genes for $\alpha = 10$.

S9. Sensitivity and ORF length

Another approach to the analysis of correlation between ORF length and sensitivity is to calculate the sensitivity across all genes at a given location, and test if these values correlate

to the gene length. In Figure S16 we can see that, not surprisingly, the highest correlation values appear in the first nine codons. Moreover, the next 9 also exhibit some significant but lower correlation. The correlation and its significance further decreases until practically zero after ~30 codons. Notice that because we discuss negative perturbations here, the sensitivity values are negative. Thus, a positive correlation means that shorter genes have a higher **absolute** sensitivity.

Figure S17 shows the same analysis for $p = +50\%$. As expected, a similar effect exists (in the opposite direction) but with more excessive noise. Specifically, the correlation barely exists after the first 9 codons.

In Figure S18 we see the same analysis for $\alpha = 10, p = -50\%$. Generally, we expect a positive correlation because shorter genes are expected to have a higher absolute sensitivity. However, we see that the first nine codons have a negative correlation. This is explained by the fact that shorter genes also tend to have higher initiation rates. When $\alpha = 10$ is considered, shorter genes "migrate" towards regime 2 and 3 (e.g. moving right in the regimes at Fig. 4A), resulting in lower absolute sensitivity. This effect has an opposite direction in terms of correlation between sensitivity and length. The first nine codons are more susceptible to changes in the initiation rate than the following 9, resulting in inverted correlation. This effect is weaker for the following 9 codons, although still exists and decreasing the correlation that is maximized around codons 30-50.

Another issue to discuss in the context of the sensitivity and length is a control of the first codons. It is important to perform and analysis of the correlation between sensitivity and length while controlling the first nine locations, in order to account for additional relations. Figure S19 shows this correlation for the representative set of 500 genes in 3 cases:

1. Overall sensitivity *vs.* ORF length
2. Start region sensitivity *vs.* ORF length (as shown for all the genes in the main text)
3. Mean sensitivity of all codons other than the first nine *vs.* ORF length

Even though a significant correlation ($r = 0.34$, $p_{value} \approx 7 \times 10^{-15}$) exists in the first case, the partial correlation while controlling the first nine codons shows that most the correlation is indeed explained by those first nine codons. This can be also seen by directly observing the correlation in the third case.

Finally, we note that as MTDR also correlated with length, we wanted to make sure this it is not a confounding factor in the current discussion. We have performed a partial spearman correlation with an MTDR control, what resulting in the same correlations. We conclude that MTDR cannot explain the observed correlation between sensitivity and ORF length.

S10. <u>Sensitivity and protein abundance</u>

In the main text, separation of the data into bins served as a type of length control. Without separating to bins, the correlation for $\alpha = 1$ is -0.37 ($P_{value} = 3.2 \times 10^{-119}$) and -0.29 ($P_{value} = 2.5 \times 10^{-70}$) for $\alpha = 10$. When performing partial Spearman correlation, while controlling the length, the result for $\alpha = 1$ is -0.35 ($P_{value} = 7.0 \times 10^{-103}$) and -0.27 ($P_{value} = 7.9 \times 10^{-61}$) for $\alpha = 10$.

MTDR is known to be correlated with PA, thus might serve as a confounding variable causing some of the observed correlation. Indeed, the partial, MTDR controlled, Spearman correlation is -0.21 ($P_{value} = 1.9 \times 10^{-38}$) for $\alpha = 1$ and -0.17 ($P_{value} = 5.5 \times 10^{-24}$) for $\alpha = 10$. Finally, we have already seen the role of ξ factor in determining the general sensitivity profile of a gene. It turns out that ξ also correlates with PA (see Figure S20): Spearman correlation is 0.38 ($p_{value} \approx 3 \times 10^{-125}$); genes with higher translation initiation rate (relative to their elongation rate) tend to have higher protein levels.

In

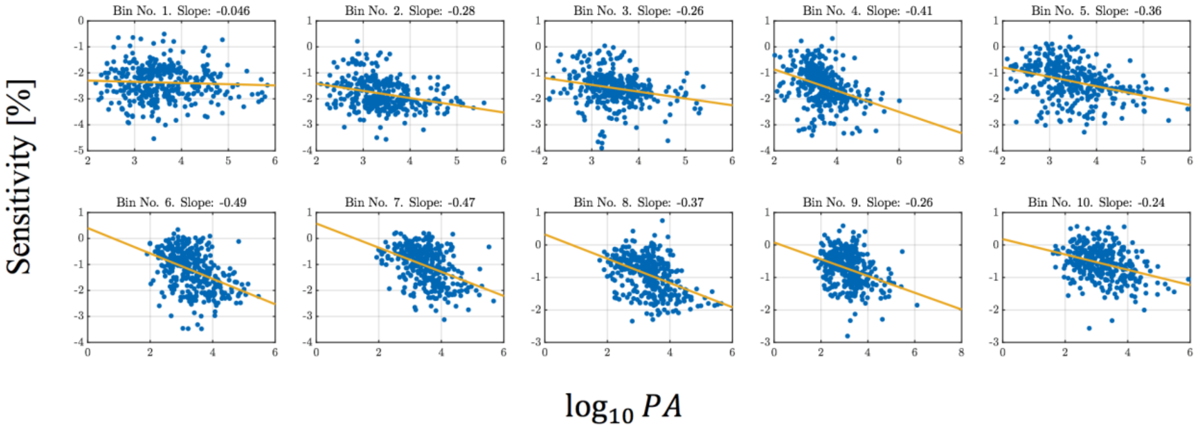

Figure S21, we see a scatter plot for each bin discussed in the main chapter, while Figure S22 summarizes the information about the slopes. Bins no. 6-8 are the most significant (even though bin 8 has a slightly lower slope), with a linear fit function of: $y = -0.44 \times \log_{10}(\text{PA}) + 0.43$ %.

Similarly, for $\alpha = 10$ and while averaging the sensitivity of all the ORF codons, we get the results shown in

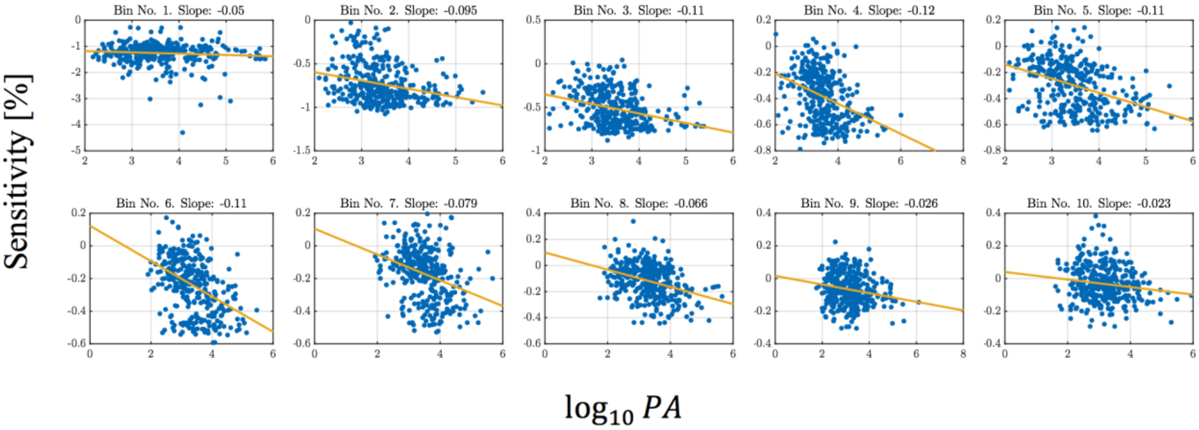

Figure S23 and Figure S24. Bin no. 6 is the most significant, with a linear fit function of: $\text{y} = -0.11 \times \log_{10}(\text{PA}) + 0.12$ %.

S11. <u>Prolonged perturbations</u>

When $\alpha = 1$, we observe a similar behavior to what previously shown. However, the sensitivity transition now occurs after codon $(s + 1 - L_p)$ where L_p is the perturbation length. In this scenario, the transition is not as steep as before, but rather takes L_p codons. This is explained by noticing that the decoding rate at the first $s = 9$ codons is what

determines the sensitivity; for a perturbation that starts among the first $(s+1-L_p)$ codons, all s first codons are perturbed, while for the next $L_p - 1$ codons there is a gradual decrease of the total change induced over the first s codons. As an example, let us consider the codons at $i = 1$ and $i = 5$ for $L_p = 1$ and for $L_p = 10$. In the case of $L_p = 1$, perturbing codon $i = 5$ effectively limits initiation as perturbing codon $i = 1$, thus their sensitivity is similar. However, when $L_p = 10$, perturbing codon $i = 1$ makes codons $i = 1$ up to $i = 10$ slower. For codon $i = 1$ this means that the initiation rate drastically reduces, because the ribosome will now be delayed at the first 10 locations in comparison to the reference state. For $i = 5$ the effective reduction is initiation rate is less severe, because only codons 5-9 will have a direct effect on initiation. For this reason, the sensitivity is lower (in absolute value) at $i = 5$ than at $i = 1$.

Furthermore, the sensitivity at these first $(s+1-L_p)$ is proportional to L_p: $\langle SP(i, -50\%)\rangle_1^{s+1-L_p} = -0.3\% - 1.1L_p$. For example, increasing L_p from 1 to 2 is expected to increase the (absolute) sensitivity by 1.1%, or relatively by 78%. The example gene at Fig. 8C shows a typical behavior. After roughly 20-30 codons, the sensitivity drops down to practically zero. The scenario of $\alpha = 10$ shows a different picture. First, the system is now less initiation limited, increasing the importance decoding rates of inner codons. When prolonged perturbations are introduced, these perturbed codons now lead to a stronger overall effect of the elongation rate, as clearly demonstrated by the example gene in Fig. 8D. Due to the fact that many genes are still initiation limited, the average profiles still exhibit increased sensitivity in the beginning. The relation between the sensitivity value and L_p is no longer linear and can be discussed for different locations i, as shown in Figure S25. For example, considering $i = 50$, increasing L_p from 1 to 2 is expected to increase the (absolute) sensitivity by 0.8%, or relatively by 158%. To conclude, prolonged perturbations increase the sensitivity in almost-linear and regime dependent manner.

S12. <u>Elongation factors perturbation</u>

For each discussed codon, we have 5,191 sensitivity values, as the number of genes. We can analyze the relation between sensitivity and elongation rate in two ways: (1) mean sensitivity values for each codon *vs.* the decoding rate (resulting in 60 data points, as presented in Figure S26); (2) all sensitivity values from all codons and genes (resulting in 60×5191 data points). When considering the original initiation rates, a significant but remarkably low correlation was found in case (2): $R_{Spearman} = -0.0066$ and $R_{Pearson} = 0.04$ (P_{value} are shown in the figure). The correlations for case (1) are not significant.

We have seen that when the baseline initiation rates values are used, the first nine codons have a dominant effect regarding the sensitivity of the system. This effect induces a bias that nullifies the correlation between sensitivity and elongation rate. Another way (in addition to what discussed in the main text) to control this effect is to perturb all the relevant codons except the first nine. Indeed, this results in a significant correlation of more than 0.3 when the mean sensitivity values per codon are considered, as seen in Figure S27.

Figure 9B showed that when group 1c (which for each codon contain all genes that do not have this codon along the first 9 codons) was considered, Spearman correlation was observed. In order to test whether this is related to codons 10-18 only or also to more distant codons, an additional group was defined: $\Gamma_4 = \Gamma(C; \{19, \dots, n\}, \{2, \dots, 18\})$. The correlation for this group is low and not significant. This means that the correlation between elongation rate and sensitivity is highly dominated by the first nine codons and they dominate the effect. High correlation is observed in the next nine codons as well, but with lower absolute sensitivity values. When ignoring the first 18 codons, no correlation observed at all; which is also the case when observing all codons altogether, emphasizing the importance of controlling the start region.

In Figure S28 we show the discussed results for other various of α. The general conclusion follows from this panel of figures is that as α increases, the difference between the different groups becomes less important, i.e. the discussed control is crucial only for the low baseline initiation rates.

S13. Functional analysis

As explained in the main text, the main approach we took in order to perform functional analysis was to look at a gene-specific sensitivity value that is calculated by perturbing all *types* of the unique codons in the gene (similarly to the elongation factor analysis), and averaging theses values. The supplementary dataset "Functional Analysis - Global Codons.xlsx" file lists all the selected sub-groups of genes, for several top and bottom percentiles of the sensitivity values. Each sheet contains the corresponding results from http://www.geneontology.org/. Results are provided for both $\alpha = 1$ and $\alpha = 10$, but as expected, more significant terms were found for higher initiation rates.

Using the same sensitivity measures for each gene, we performed another analysis using slim-GO terms from https://downloads.yeastgenome.org/curation/literature/go_slim_mapping.tab. This time, only for $\alpha = 10$, we compared median sensitivity value of genes that are associated with a specific term to the median sensitivity of all genes, using Wilcoxon signed rank test. Figure S32, Figure S33 and Figure S34 show the cumulative distributions of sensitivities of terms with significantly different sensitivity (whether higher or lower) with $P_{value} < 1 \times 10^{-150}$, for all 3 ontologies. All the significant terms at a 1% level with Bonferroni correction are listed in the supplementary dataset "Functional Analysis - GO_slim_Wilcox.xlsx" file. Due to differences in the set of genes, not all genes that were listed in the slim-GO database were found in our set of 5,191 genes. The number of genes found is indicated in the file.

Another approach that was considered but not discussed in the main text is the functional analysis based on the sensitivity profile. Understanding the different sensitivity regimes suggests that functional gene analysis, based on their sensitivity profile, should involve some differentiation based in the sensitivity in different regions. We suggest to characterize each gene by its start region sensitivity: $\langle SP \rangle_{i=1}^{9}$, and by the average sensitivity at all the other codons: $\langle SP \rangle_{i=10}^{end}$. Now we can define 4 mutually exclusive groups: First group contains genes with the 50% lowest (absolute) values of $\langle SP \rangle_{i=1}^{9}$ AND 50% lowest values of $\langle SP \rangle_{i=10}^{end}$

and will be denoted ‘low_all_low_f9’. In similar way, we define ‘low_all_hi_f9’, ‘hi_all_low_f9’ and ‘hi_all_hi_f9’ (see Figure S35A). It is also possible to define similar groups with more strict thresholds of, for example, 20%. These groups will be symbolized with the addition of a letter ‘D’ (see Figure S35B). In this approach, only $\alpha = 10$ is presented, as baseline initiation rates resulted in no significant results.

The supplementary dataset “Functional Analysis - Profile Based.xlsx” file lists all the genes in each one of the 8 defined groups, and the corresponding results from http://www.geneontology.org/.

Below a summary of the analysis parameters for both profile-based and global codons analysis:

- Analysis Type: PANTHER Overrepresentation Test (Released 20171205)
- Annotation Version and Release Date - GO Ontology database Released 2017-12-27
- Reference List – The set of 5,191 genes mentioned in the main text.
- Annotation Data Set - PANTHER Pathway
    - PANTHER Protein Class;
    - GO molecular function complete;
    - GO biological process complete;
    - GO cellular component complete.
- Test Type - Fisher's exact test with FDR correction

Supplementary Figures

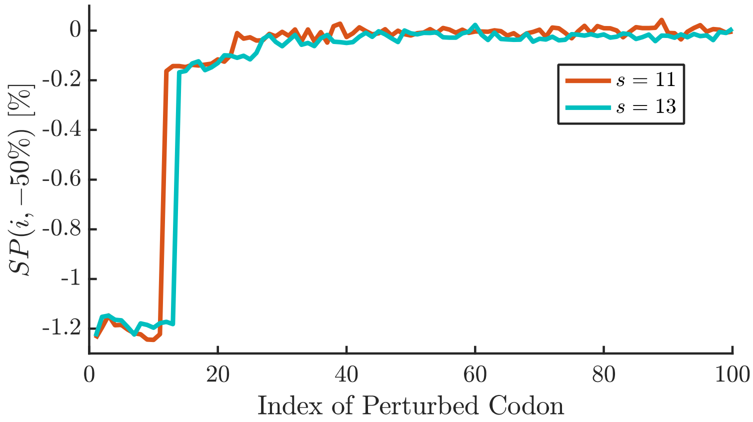

Figure S1: Sensitivity profiles for ~500 representative genes, using 11 and 13 codons length ribosomes. We see that the sensitivity value itself does not change, but only the sensitive region (which corresponds to the ribosome length, as expected).

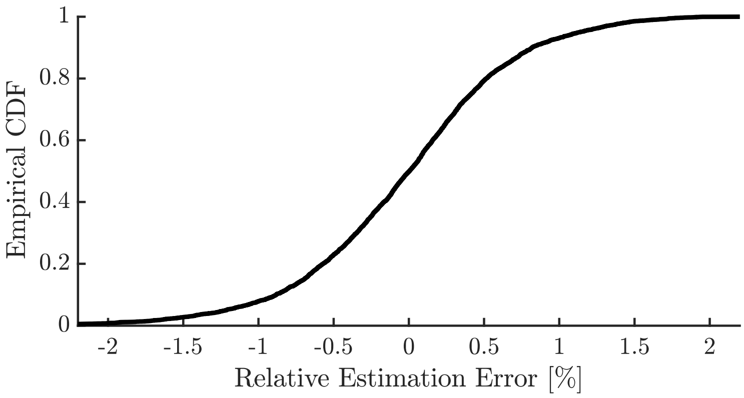

Figure S2: Distribution of errors in estimating initiation rates of 5,191 yeast genes. Parameter estimation for a normal distribution results in $\hat{\mu} = -0.02\%$ (std. error of 0.01%) and $\hat{\sigma} = 0.75\%$ (std. error of 0.01%).

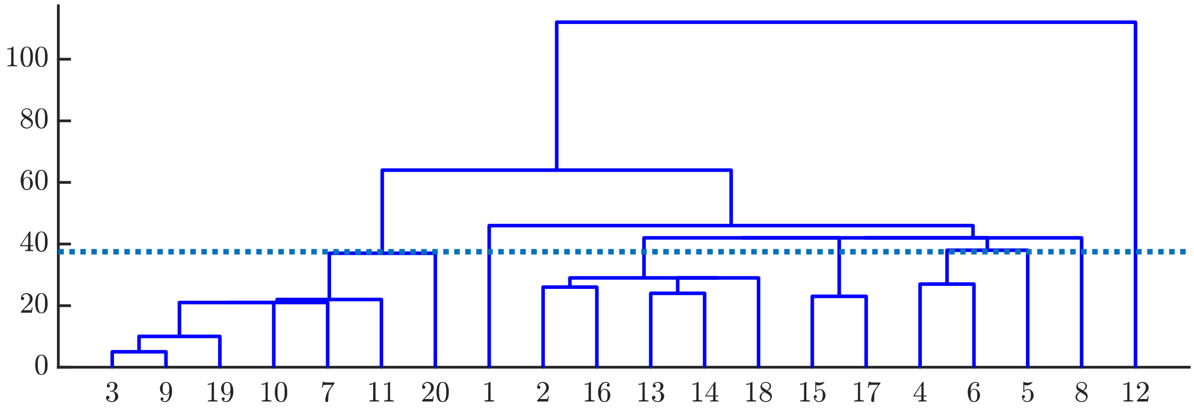

Figure S3: Dendrogram of the used hierarchal clustering. Each cluster contains similar amino acids according to the Grantham[2] metric.

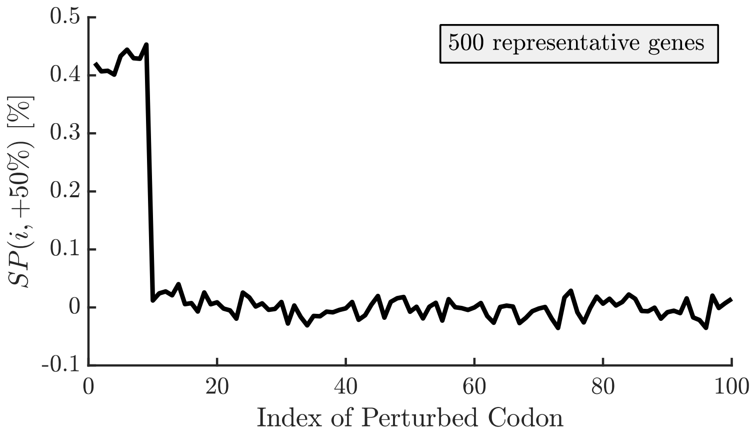

Figure S4: Average sensitivity profile of 500 representative gens with positive perturbations of 50% (i.e. the codons are faster). Similar behavior to the negative perturbations is observed but in opposite direction and lower magnitude.

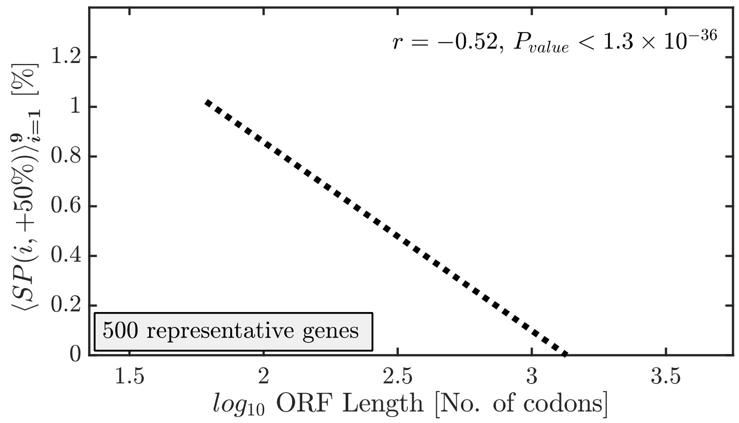

Figure S5: Start region sensitivity (first nine codons) *vs.* the ORF length with positive perturbations of 50%. A linear estimation results in: $y = -0.76 \log_{10}(length) + 2.38\%$.

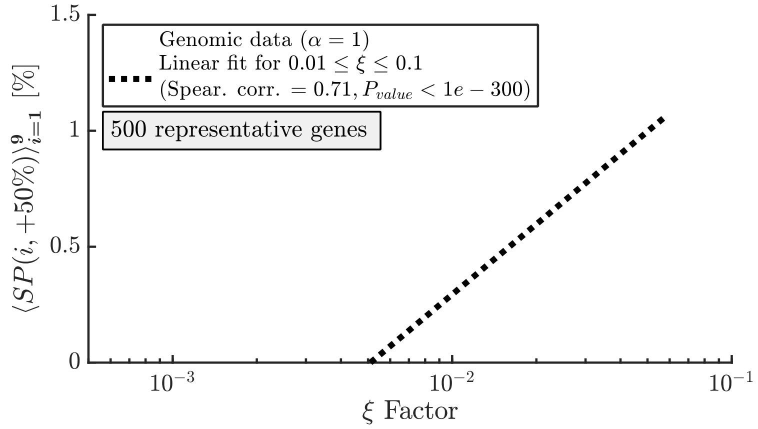

Figure S6: Start region sensitivity *vs.* ξ with positive perturbations of 50%. The correlation was calculated for the range of $0.01 \leq \xi \leq 0.1$ and so as a linear fit: $y = \log_{10} \xi + 2.3\%$.

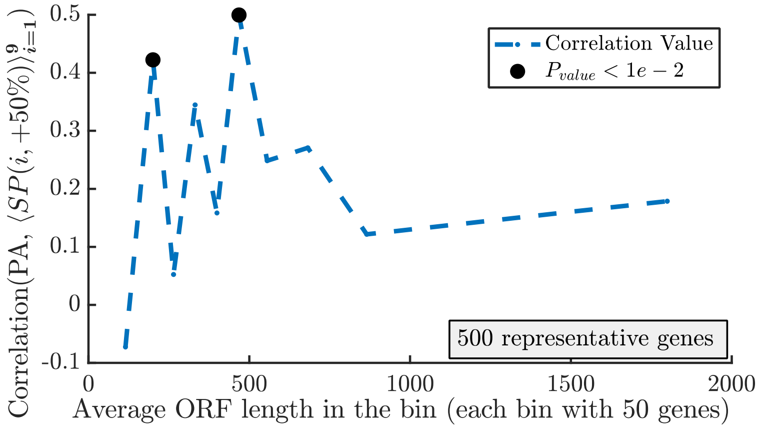

Figure S7: Spearman correlation between start region sensitivity and protein abundance, divided into 10 bins with genes of similar ORF length, with positive perturbations of 50%. The result resembles what we have seen for negative perturbation, but again, with a smaller effect (which also results in less significant correlations).

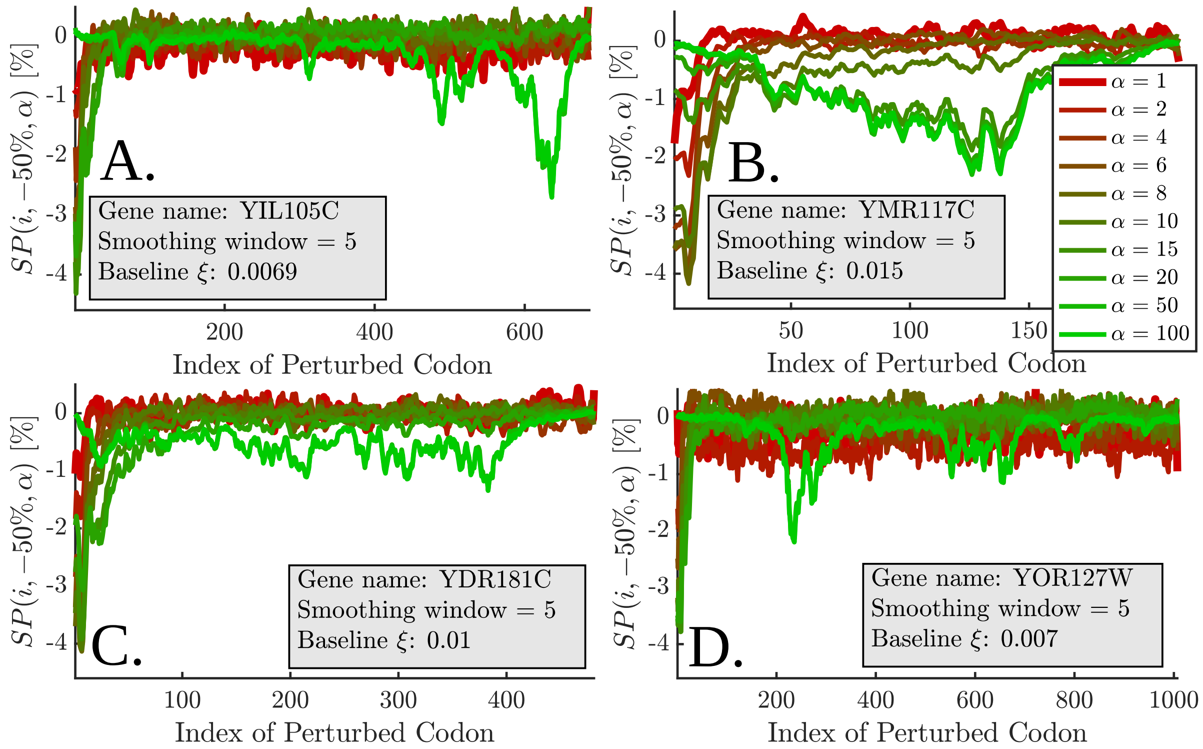

Figure S8: Several examples of genes and their sensitivity profile for different. In all cases similar behavior appears: high α values give rise to sensitivity at various codons inside the ORF, while low α result in profiles with high sensitivity at the start region, and low sensitivity anywhere else.

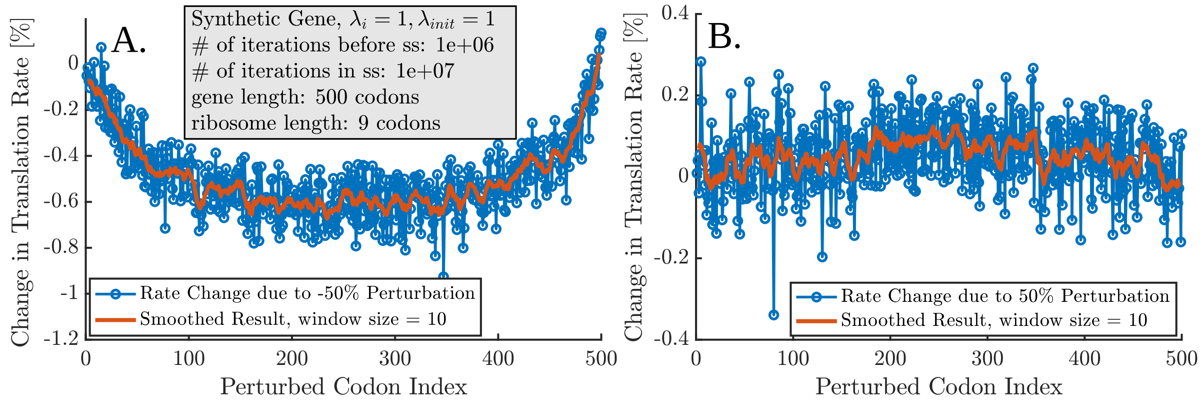

Figure S9: Canonical examples of sensitivity profiles of synthetic gene with default parameters. A. negative perturbation. B. positive perturbation.

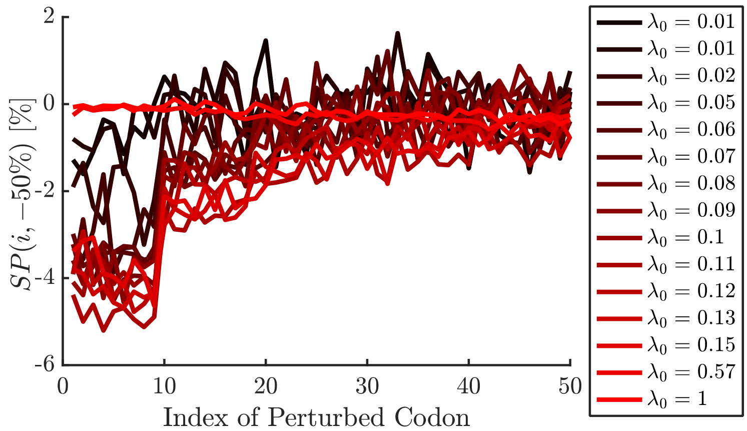

Figure S10: Sensitivity profiles of synthetic gene with several low values of initiation rate. It can be seen that the sensitivity in the first nine codons is higher relative to the rest of the ORF, and that there is some non-trivial relation between the sensitivity at this region and the initiation rate.

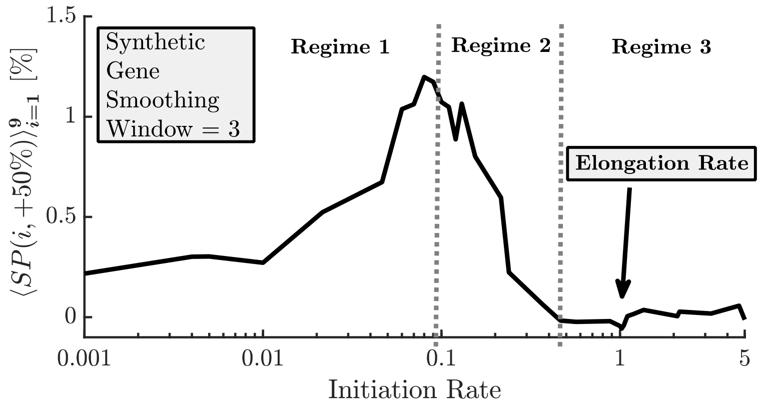

Figure S11: Regimes diagram, which is the start region sensitivity *vs.* initiation rate. This result was obtained for synthetic gene with positive perturbation of 50%.

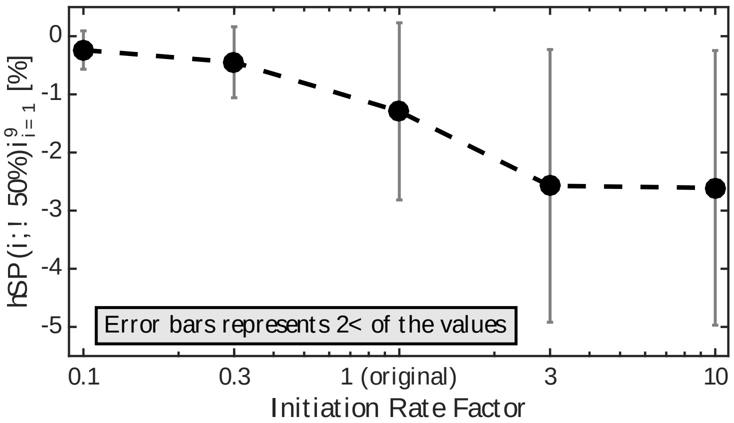

Figure S12: Start region sensitivity *vs.* initiation rate, up to $\alpha = 10$.

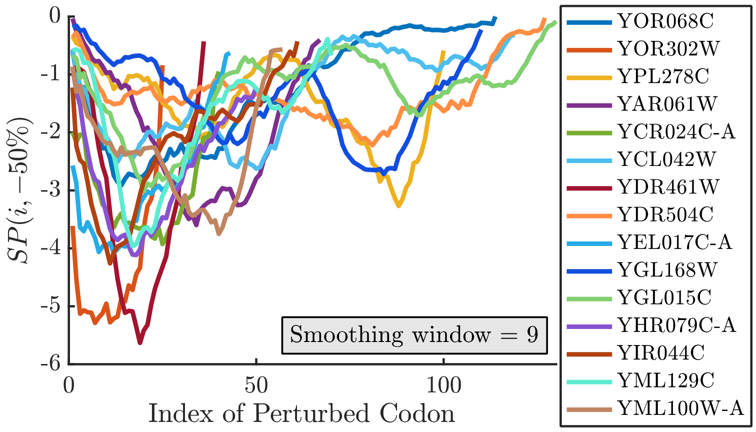

Figure S13: Sensitivity profiles of the 15 genes that satisfy $\xi \geq 0.5$ for baseline initiation rates. It can be seen that these profiles do not exhibit the increased sensitivity at the start region, but rather have sensitive regions at various position along the ORF.

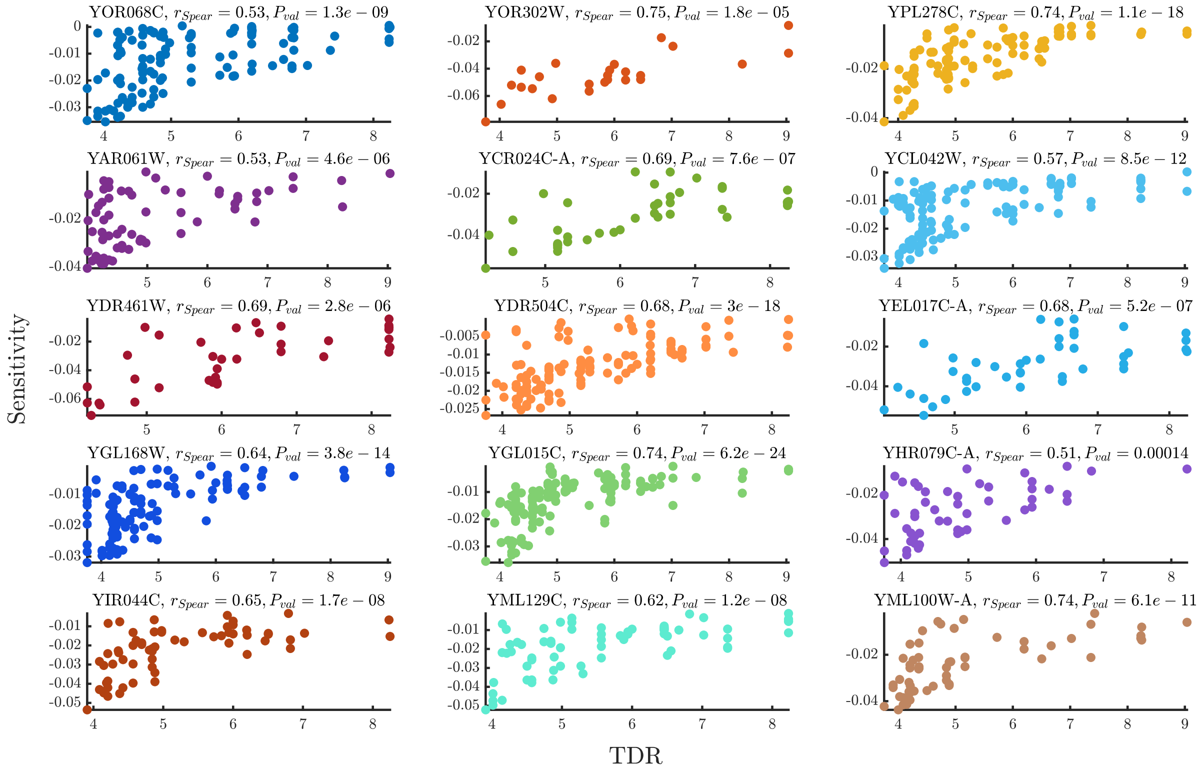

Figure S14: Sensitivity of codons *vs.* their TDR (typical decoding rate) for the 15 genes that satisfy $\xi \geq 0.5$ for baseline initiation rates. Each point represents a single codon at a the ORF. All correlations are relatively high and significant.

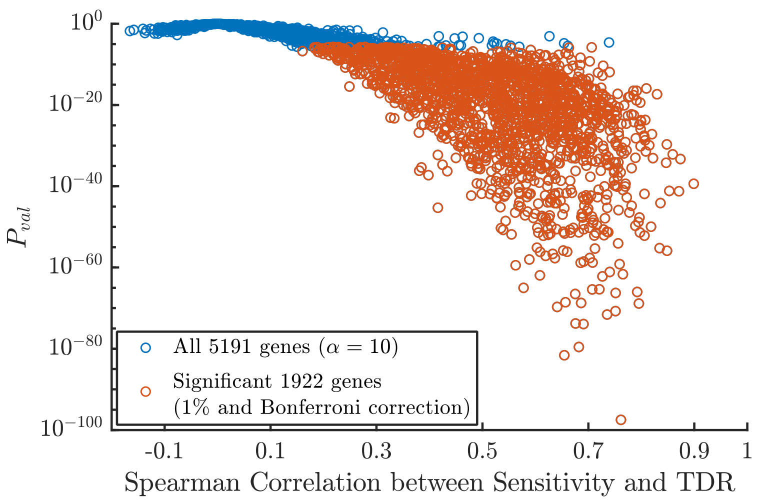

Figure S15: P_{value} *vs.* the correlation value between sensitivity and TDR. This demonstrates that a large portion of the discussed genes exhibit such a relation between the sensitivity and the TDR when initiation rate is increased.

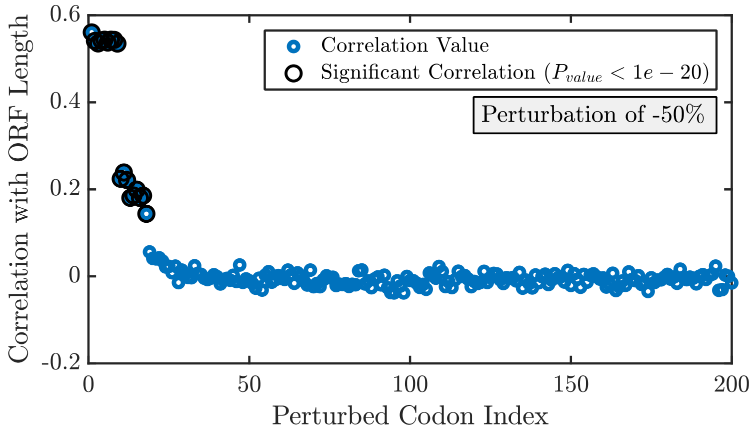

Figure S16: Spearman correlation between sensitivity at a specific location (averaged across all the discussed genes) and the ORF length in codons for a -50% perturbation. This result shows that individual codons along the start region also exhibit increased sensitivity.

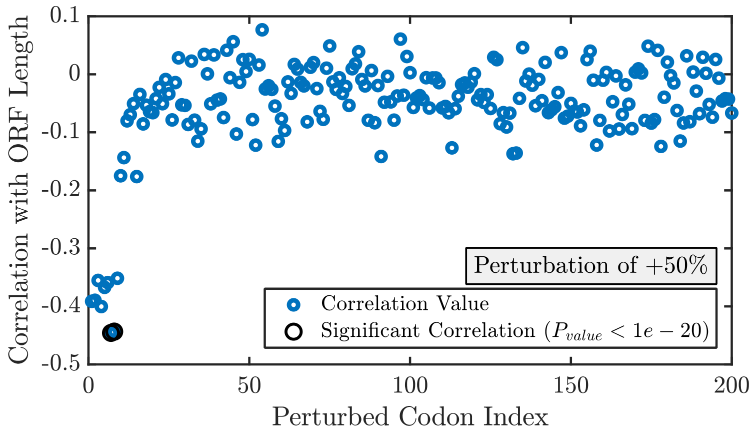

Figure S17: Similar to Figure S16 but for a positive +50% perturbation. Correlations are lower to the smaller sensitivity (and thus higher signal to noise ratio).

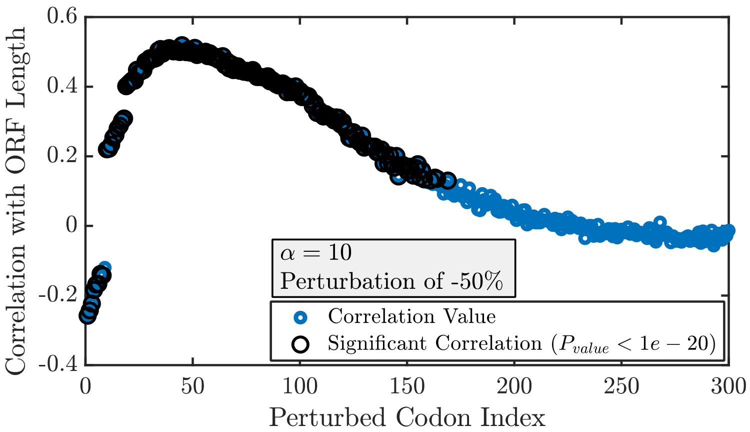

Figure S18: Spearman correlation between sensitivity at a specific location and the ORF length in codons, -50% perturbation and $\alpha = 10$. Interestingly, in contrary to Figure S16, the correlation at the first codons is negative. A possible explanation is discussed in supplementary results S9.

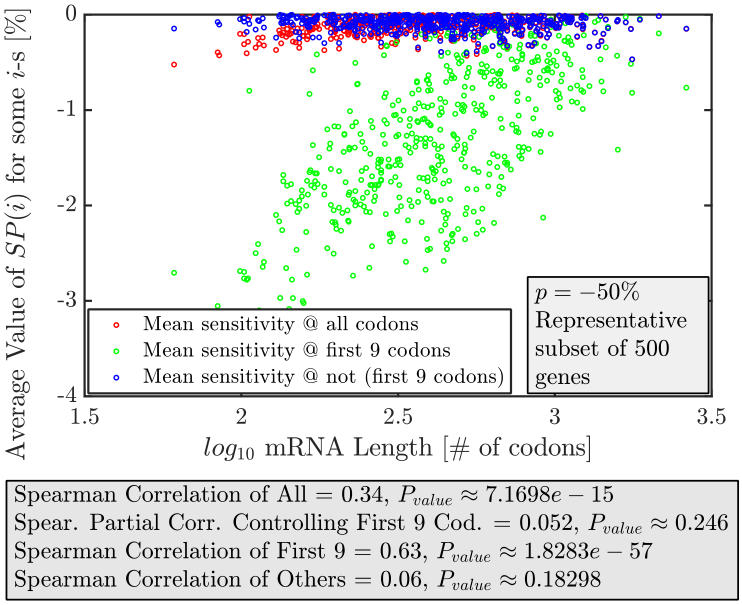

Figure S19: Average sensitivity *vs.* ORF length for several sub-groups of indexes. This result shows that even though overall sensitivity correlates with the length, most of the signal comes from the start region.

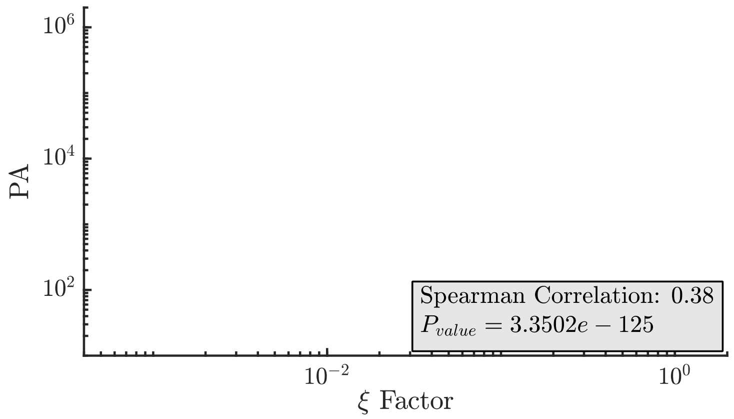

Figure S20: Protein abundance *vs.* ξ factor

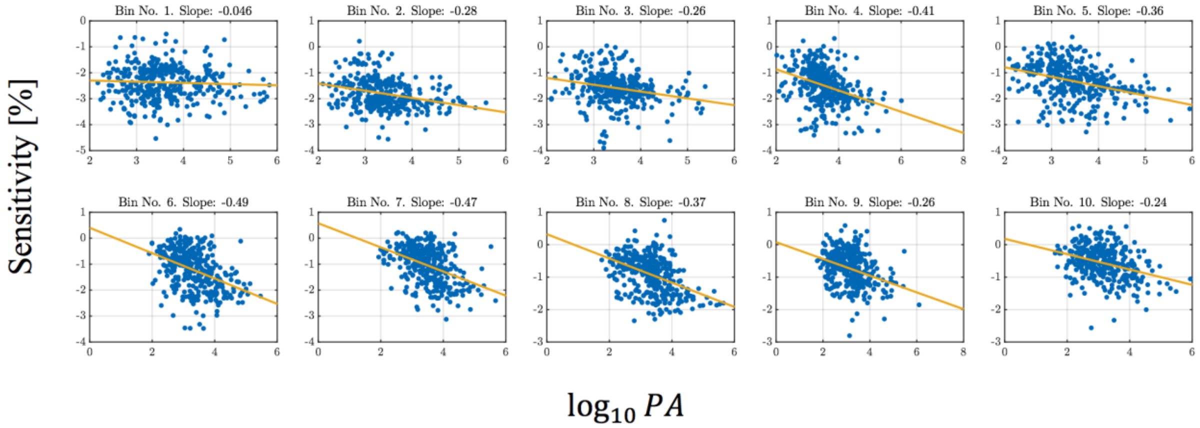

Figure S21: Start region sensitivity *vs.* $\log_{10} PA$ for each bin separately, for $\alpha = 1$. Each bin contain genes with similar ORF length.

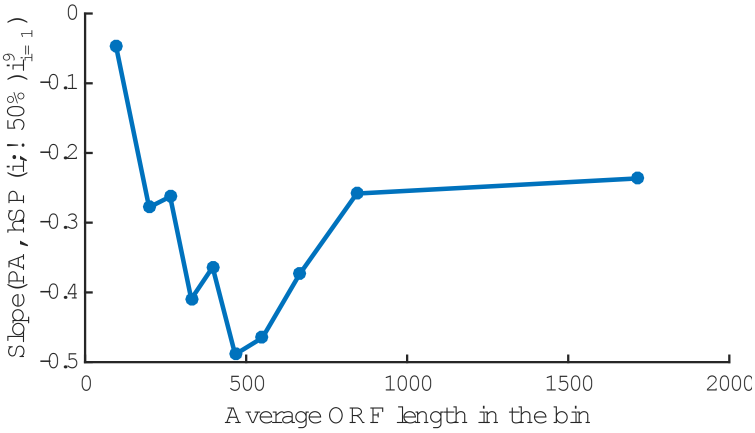

Figure S22: Slope of the linear fit between sensitivity (first nine codons, $\alpha = 1$) and PA for each of the 10 bins. Bins no. 6 and 7 have the steepest slope.

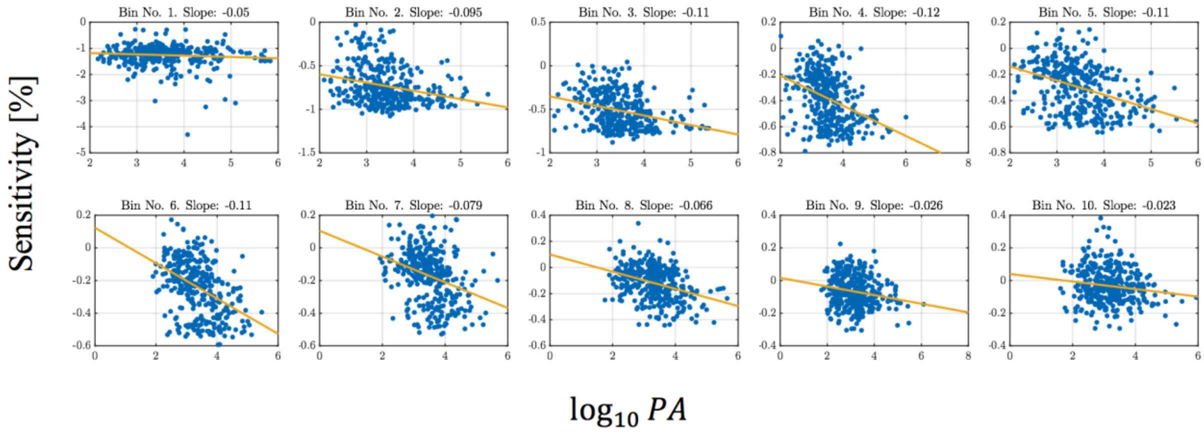

Figure S23: Start region sensitivity *vs.* $\log_{10} PA$ for each bin separately, for $\alpha = 10$.

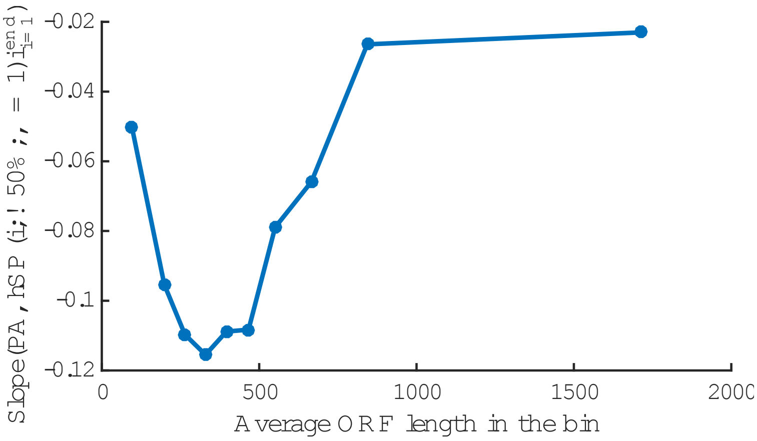

Figure S24: Slope of the linear fit between sensitivity (all codons, $\alpha = 10$) and PA for each of the 10 bins. Bins no. 3-6 have the steepest slope.

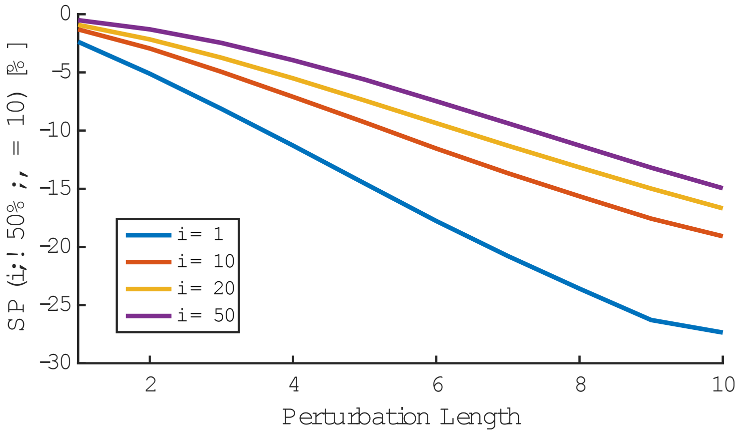

Figure S25: Sensitivity *vs.* perturbation length (in codons) for several locations. Every color represents a specific location.

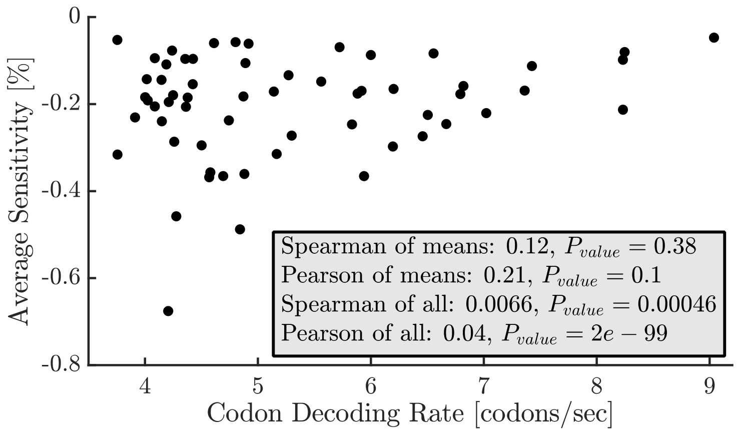

Figure S26: Overall sensitivity *vs.* codon decoding rate, when global elongation factors are perturbed (perturbation of -50%). Each point represents the average sensitivity of a specific codon types across all the genes (represented in the text-box as ‘means’). Alternative analysis would be to take 5,191 values for each such codons (one value per gene), resulting in slightly different correlations (represented in the text-box as ‘all’).

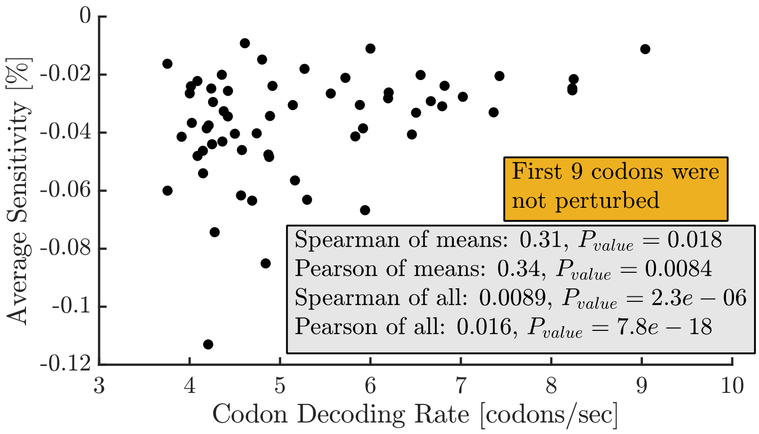

Figure S27: Overall sensitivity *vs.* codon decoding rate, when global elongation factors are perturbed (perturbation of -50%), but this time the first 9 codons were not perturbed in order to control their contribution to sensitivity.

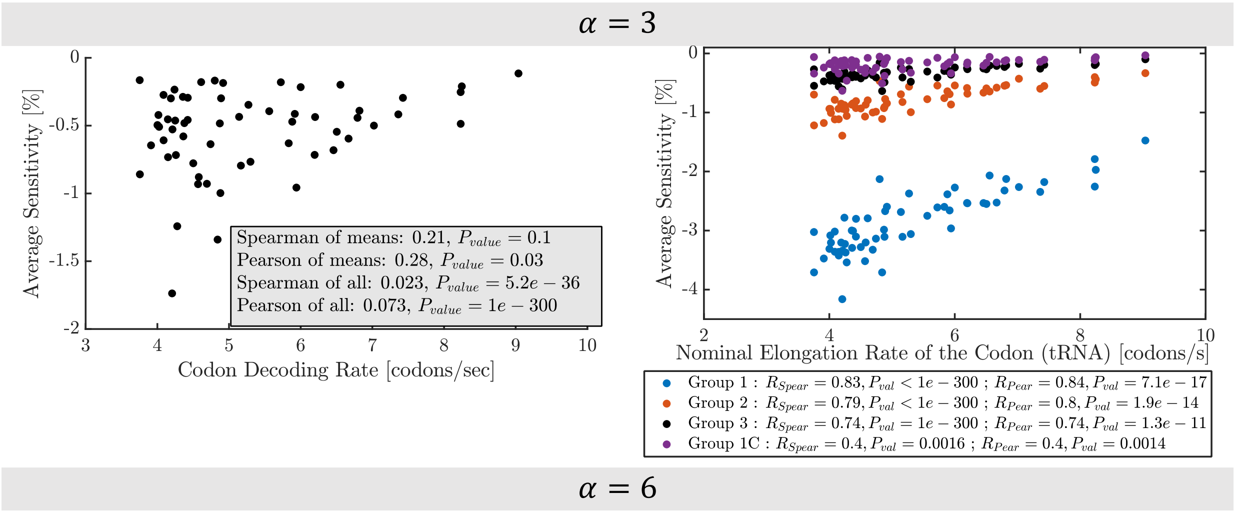

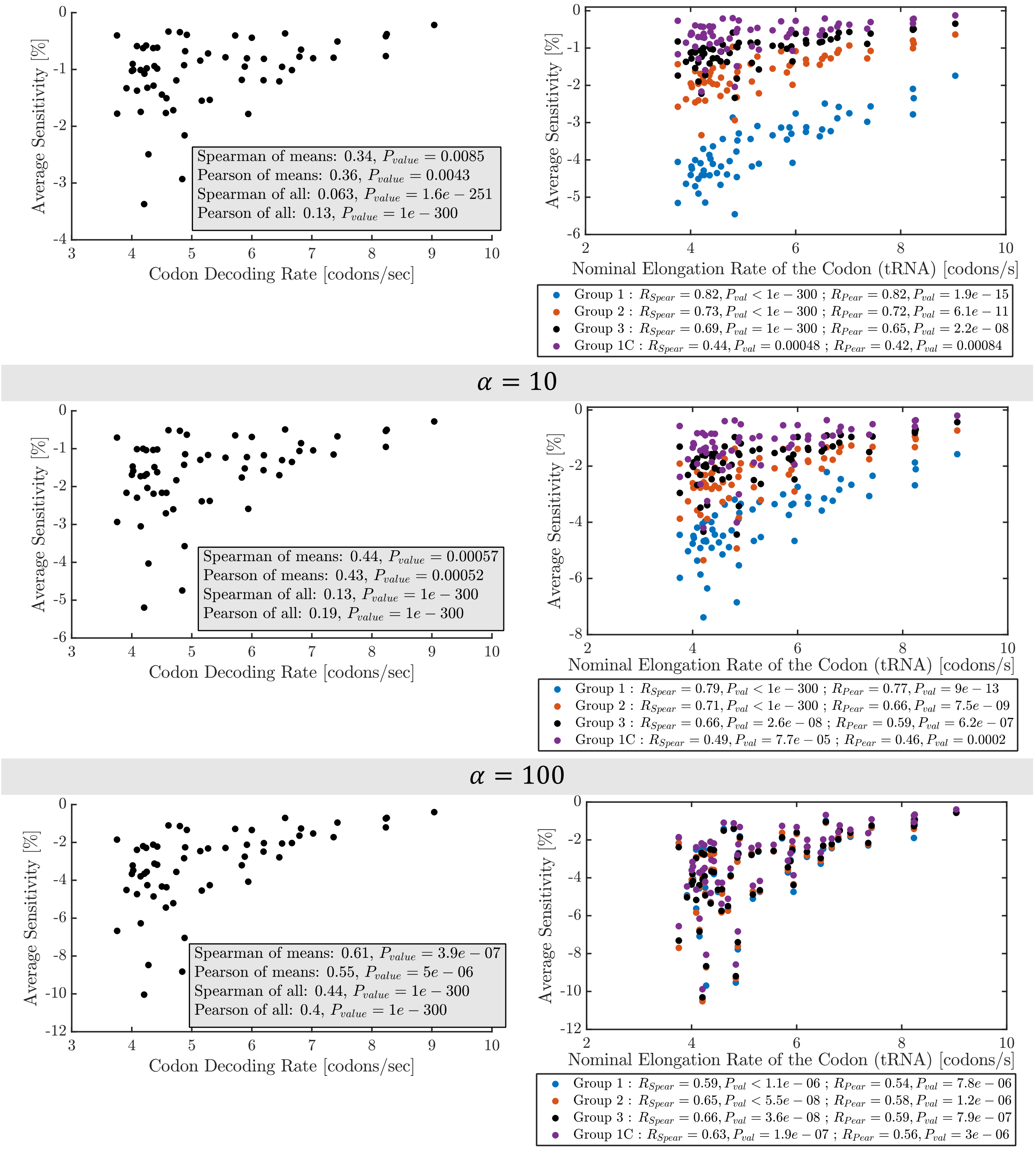

Figure S28: Panel showing overall sensitivity *vs*. decoding rate for all genes (left) and same thing for the 4 groups defined in the main text (right) for $\alpha = 3,6,10$ and 100.

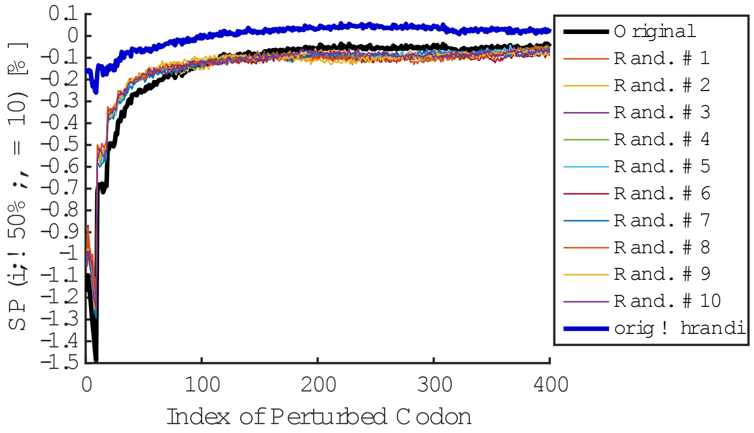

Figure S29: Average sensitivity profiles of the original genes and the 10 variants of mutations of the first type. The thick blue line is the difference. For the first ~50 codons, the original genes tend to be more sensitive. For example, the average original sensitivity in codons 1-9 is -1.28%, while for the random variants it is -1.12%. This is an absolute difference of 0.16% or relative difference of roughly 14%. On the other hand, in more distant codons the randomized genes tend to be more sensitive. For example, in codons 200-300, the average sensitivity of the original genes is -0.053%, while for the randomized it is -0.096%, thus an absolute difference of 0.043% or roughly a 56% relative change.

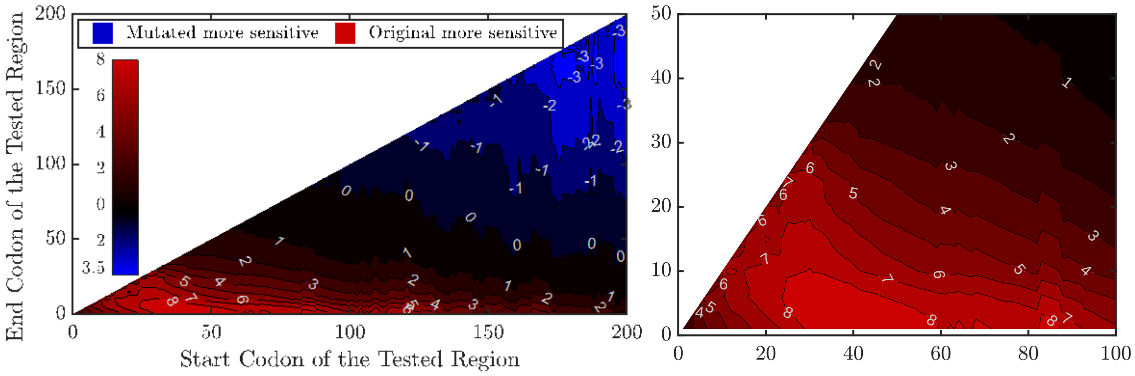

Figure S30: significance map ($\Pi_{a,b} = -10\log_{10} P_{val}$, as defined in the main text) of the difference between the original and the mutative sensitivity profiles, for the second type of mutations. The negative signs among the “blue” region are to be ignored.

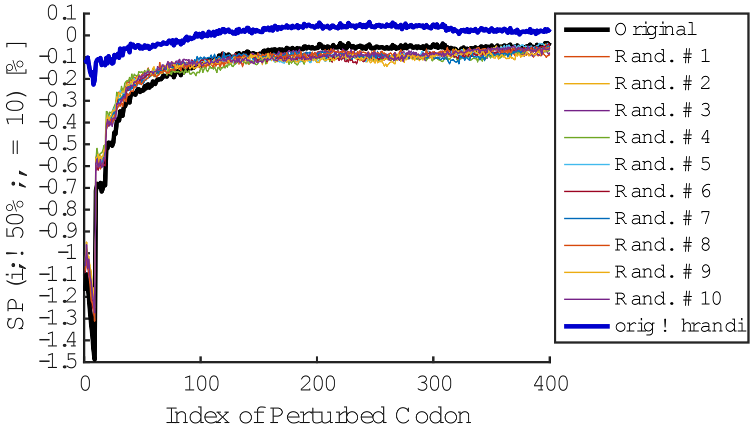

Figure S31: Average sensitivity profiles of the original genes and the 10 variants of mutations of the second type. The thick blue line is the difference. The values are very similar (but not identical. See, for example, the first values of the difference graph) to what shown in Figure S30.

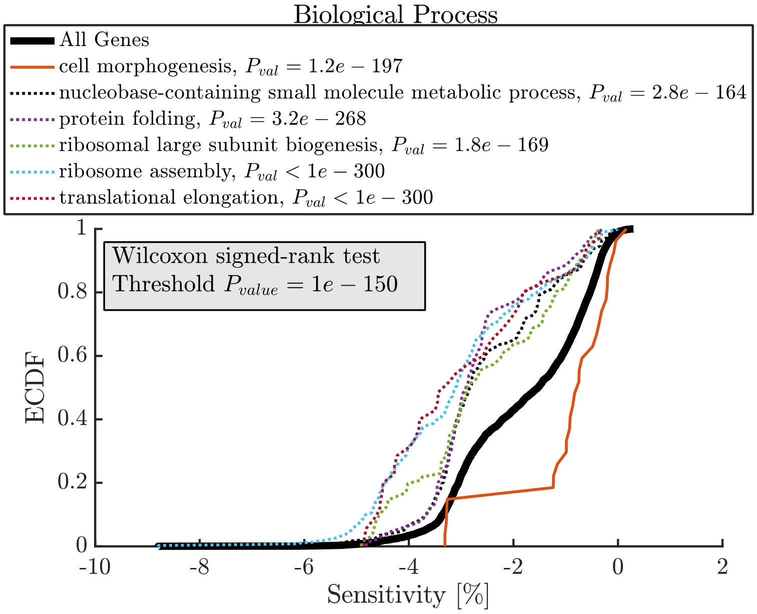

Figure S32: CDF of the sensitivity values of group of genes that have significantly different mean that all the sensitivity values. Each gene group is related to a specific ontology term. Here the 'Biological Process' is shown.

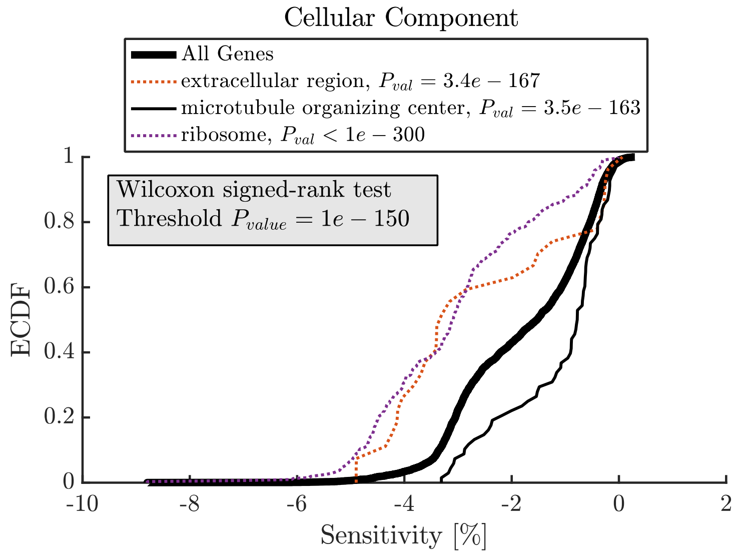

Figure S33: CDF of the sensitivity values of group of genes that have significantly different mean that all the sensitivity values. Each gene group is related to a specific ontology term. Here the ‘Cellular Component’ is shown.

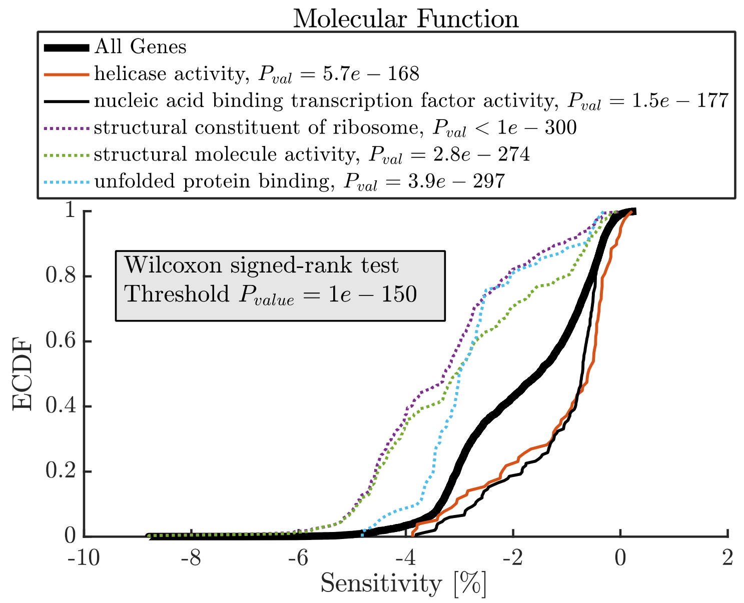

Figure S34: CDF of the sensitivity values of group of genes that have significantly different mean that all the sensitivity values. Each gene group is related to a specific ontology term. Here the ‘Molecular Function’ is shown.

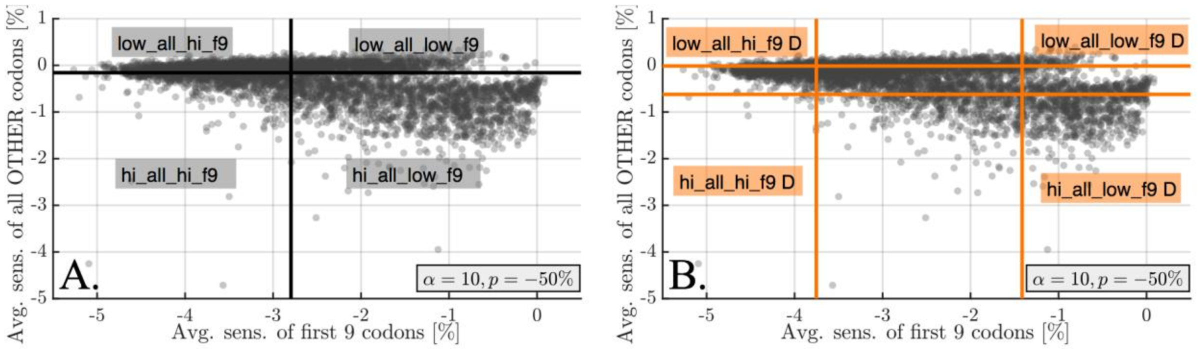

Figure S35: Visualization of the different groups used for functional analysis in the approach of sensitivity profile-based features. Each gene was characterized by its start region sensitivity and the average sensitivity at the rest of the codons. Enrichment was tested at each group separately.

Supplementary Tables

Table S1: Details of the 8 clusters used for clustering the second type of mutations. The number at the column '#' corresponds to the node of the dendrogram in Figure S3.

Cluster	Amino Acid	#	Amino Acid	Code	Hydropathy	Charge	pKa, NH2	pKa, COOH	pK(R)	Solubility
1	Thr	5	Threonine	T	hydrophilic	N	9.12	2.15		freely
2	Ala	6	Alanine	A	hydrophobic	N	9.87	2.35		15.8
2	Pro	4	Proline	P	hydrophobic	N	10.6	1.99		1.54
3	Asn	15	Asparagine	N	hydrophilic	N	8.8	2.02		2.4
3	Asp	17	Aspartate	D	hydrophilic	-	9.6	1.88	3.65	0.42
4	Arg	2	Arginine	R	hydrophilic	+	9.09	2.18	13.2	71.8
4	Gln	14	Glutamine	Q	hydrophilic	N	9.13	2.17		2.6
4	Glu	18	Glutamate	E	hydrophilic	-	9.67	2.19	4.25	0.72
4	His	13	Histidine	H	moderate	+	8.97	1.78	6	4.19
4	Lys	16	Lysine	K	hydrophilic	+	10.28	8.9	2.2	
5	Gly	8	Glycine	G	hydrophobic	N	9.6	2.34		22.5
6	Ser	1	Serine	S	hydrophilic	N	9.15	2.21		36.2
7	Ile	9	Isoleucine	I	hydrophobic	N	9.76	2.32		3.36
7	Leu	3	Leucine	L	hydrophobic	N	9.6	2.36		2.37
7	Met	19	Methionine	M	moderate	N	9.21	2.28		5.14
7	Phe	10	Phenylalanine	F	hydrophobic	N	9.24	2.58		2.7
7	Trp	20	Tryptophan	W	hydrophobic	N	9.39	2.38		1.06
7	Tyr	11	Tyrosine	Y	hydrophobic	N	9.11	2.2	10.1	0.038
7	Val	7	Valine	V	hydrophobic	N	9.72	2.29		5.6
8	Cys	12	Cysteine	C	moderate	N	10.78	1.71	8.33	freely

References

1. Arava, Y. *et al.* Genome-wide analysis of mRNA translation profiles in Saccharomyces cerevisiae. *Proc. Natl. Acad. Sci. U. S. A.* **100,** 3889–3894 (2003).

2. Grantham, R. Amino acid difference formula to help explain protein evolution. *Science (80-.).* (1974). doi:10.1126/science.185.4154.862
